# Fixation probabilities in graph-structured populations under weak selection

**DOI:** 10.1371/journal.pcbi.1008695

**Published:** 2021-02-02

**Authors:** Benjamin Allen, Christine Sample, Patricia Steinhagen, Julia Shapiro, Matthew King, Timothy Hedspeth, Megan Goncalves

**Affiliations:** Department of Mathematics, Emmanuel College, Boston, Massachusetts, United States of America; Max-Planck-Institute for Evolutionary Biology, GERMANY

## Abstract

A population’s spatial structure affects the rate of genetic change and the outcome of natural selection. These effects can be modeled mathematically using the Birth-death process on graphs. Individuals occupy the vertices of a weighted graph, and reproduce into neighboring vertices based on fitness. A key quantity is the probability that a mutant type will sweep to fixation, as a function of the mutant’s fitness. Graphs that increase the fixation probability of beneficial mutations, and decrease that of deleterious mutations, are said to amplify selection. However, fixation probabilities are difficult to compute for an arbitrary graph. Here we derive an expression for the fixation probability, of a weakly-selected mutation, in terms of the time for two lineages to coalesce. This expression enables weak-selection fixation probabilities to be computed, for an arbitrary weighted graph, in polynomial time. Applying this method, we explore the range of possible effects of graph structure on natural selection, genetic drift, and the balance between the two. Using exhaustive analysis of small graphs and a genetic search algorithm, we identify families of graphs with striking effects on fixation probability, and we analyze these families mathematically. Our work reveals the nuanced effects of graph structure on natural selection and neutral drift. In particular, we show how these notions depend critically on the process by which mutations arise.

## Author summary

When a new mutation appears in a population, it may ultimately spread to all individuals, or it may go extinct. Which outcome occurs depends on how the mutation affects the organism’s fitness (i.e., natural selection), but also on random chance. The spatial arrangement of organisms in the population can alter the balance between selection and random chance: amplifying one, suppressing the other. However, these effects can be difficult to predict or compute, even in simple, idealized mathematical models. We develop a method to efficiently calculate the effects of spatial structure on natural selection, in the case that mutations have only a weak effect on fitness. We use this method to comb through millions of distinct spatial structures, identifying those with the most extreme effects on natural selection. The question we study has applications to cancer research, with an individual’s cells considered as a population, of which cancer cells are an invading mutant type.

## Introduction

Evolution proceeds by the arrival and fixation of mutations. The fate of each new mutation depends on selection (how the mutation affects the organism’s fitness) as well as drift (random chance). The combined effects of selection and drift determine how a population evolves, with selection driving adaptation to the environment, and drift maintaining genetic variety.

Spatial population structure can alter the balance between these two forces [1–13]. Some spatial structures *amplify* selection, so that fitness plays a larger role in which mutations become fixed. Other structures *suppress* selection, reducing the role of fitness and increasing the role of random drift. Spatial structure can also change the accumulation rate of neutral mutations, which do not affect fitness [14]. The effects of spatial structure on selection have consequences for microbial evolution [15], cancer [16–20], aging [19, 20], and infectious disease [21].

These effects can be probed mathematically by modeling population structure as a graph. Each vertex is occupied by a single haploid individual. Reproduction occurs along the graph’s edges, according to a specified update rule (see below). The key quantity of interest is the fixation probability *ρ*(*r*), defined as the probability that a new mutation of fitness *r* will take over a population of wild-type fitness 1.

The update rules most commonly considered in this context are Birth-death (Bd) and death-Birth (dB). In this naming convention, the ordering indicates which event (birth or death) occurs first, while the capitalization indicates which event(s) are affected by fitness (birth, in this case). Most studies [1, 3–5, 8–11, 22–26] focus on Birth-death updating, in which an individual is first selected to reproduce, proportionally to its fitness. The offspring then replaces a neighbor chosen at random (independently of fitness). A minority of works [2, 6, 12, 13, 27–29] have considered death-Birth updating, in which an individual is first selected for death, uniformly at random. A neighbor is then chosen, proportionally to fitness, to produce an offspring, which fills the vacancy.

Determining fixation probability on an arbitrary graph is computationally intensive. Current methods [6, 9, 30, 31] require solving a system of linear equations whose size grows exponentially with the graph size. This is prohibitive except for graphs that are small [6, 7, 9–11, 31], highly symmetric [1, 3–5, 22–25], or have other special properties [32].

However, most nonlethal biological mutations are either neutral (*r* = 1) or weakly selected (*r* ≈ 1). Our previous work [12, 14, 33] has shown that, in these cases, fixation probabilities can be computed in polynomial time. For neutral mutations, the fixation probability determines the population’s “molecular clock”—the rate at which neutral genetic substitutions accrue over time. Allen et al. [14] showed that spatial structure can either accelerate or slow this molecular clock rate.

For weakly selected mutations, fixation probabilities can be computed by combining perturbative methods [33–37] with coalescing random walks [38–41]. Allen et al. [12] applied this method to dB updating, and showed how it allows for the efficient identification of amplifiers and suppressors of weak selection.

Here we apply these methods to Birth-death updating on arbitrary weighted graphs. We show that fixation probabilities under weak selection can be expressed in terms of *coalescence times*—the expected times for two independent random walks to meet. These coalescence times—and hence the fixation probability for weak selection—can be computed in polynomial time. While our methods apply to arbitrary placement of the initial mutant, we focus in particular on temperature initialization (mutations arise only in new offspring) and uniform initialization (mutations arise uniformly in all individuals).

Using these methods, we compute weak-selection fixation probabilities for all simple connected graphs up to size 10. For larger sizes, we employ a previously-developed genetic algorithm [10] to identify graphs with extreme effects on fixation probability. These investigations reveal a family of “Cartwheel” graphs, which strongly amplify selection under temperature initialization. They also show that a family of “Detour” graphs [10] can significantly decrease the ratio of beneficial to neutral mutations accruing over time. Our results highlight previously-unexamined subtleties in the notions of amplifier and suppressor, and in the way that spatial structure affects neutral and selective genetic change.

## Methods

### Graph structure

We represent spatial structure by a weighted graph *G*. The edge weight from vertex *i* to *j* is *w*_*ij*_ ≥ 0. The graph may be directed (*w*_*ij*_ not necessarily equal to *w*_*ji*_) and may contain self-loops (*w*_*ii*_ may be positive). We require that the graph is *strongly connected*, meaning that there is a path of directed edges with nonzero weight from any vertex to any other; for undirected graphs (*w*_*ij*_ = *w*_*ji*_), this reduces to the usual notion of connected.

We define the *weighted (out-)degree* of vertex *i* as *w*_*i*_ = ∑_*j*∈*G*_ *w*_*ij*_. The probability that a random walker at vertex *i* will step to vertex *j* is *p*_*ij*_ = *w*_*ij*_/*w*_*i*_. We also define the *temperature* of vertex *i* as *T*_*i*_ = ∑_*j*∈*G*_ *p*_*ji*_. We note that the total temperature is equal to the total population size: ∑_*i*∈*G*_ *T*_*i*_ = ∑_*i*,*j*∈*G*_ *p*_*ji*_ = *N*.

### Birth-death process

We consider a well-studied model of natural selection [1–11, 22–30]. There are two types of individuals: residents (or wild-types), which have fitness 1, and mutants, which have fitness *r*. The mutant is advantageous if *r* > 1, deleterious if *r* < 1, and neutral if *r* = 1.

In each state of the process, each vertex is occupied by a single individual, either mutant or resident. Selection proceeds according to the Birth-death (Bd) update rule. First, an individual *i* is selected at random, proportionally to its fitness. The chosen individual *i* produces an offspring; this offspring replaces another individual *j*, chosen with probability *p*_*ij*_. Offspring inherit the type of the parent.

This process is a finite Markov chain with two absorbing states: one in which only residents are present, and one in which only mutants are present.

### Initialization

The key biological question is, if a new mutation arises in a single individual, how likely is this type to take over the resident population? The answer depends on where the initial mutant arises. In the general case, we can consider an arbitrary probability distribution {*μ*_*i*_} over the vertices of *G*, such that the initial mutation arises at vertex *i* with probability *μ*_*i*_.

Most previous works [1, 3, 4, 6, 7, 9, 10, 22–25, 30] consider only *uniform initialization*, meaning that the mutant is equally likely to appear at each vertex, *μ*_*i*_ = 1/*N* for all *i*. Uniform initialization corresponds to a biological assumption that heritable mutations arise primarily in adult individuals, with constant probability per unit time.

If we instead suppose that mutations occur primarily in new offspring, then mutations will be more likely to arise in sites that are replaced more often. This leads to *temperature initialization* [5, 8, 11, 14, 42], meaning that the initial mutant’s location *i* is chosen proportionally to the temperature *T*_*i*_, *μ*_*i*_ = *T*_*i*_/*N*.

Here we consider both initialization schemes, with a particular focus on temperature initialization because it leads to a rich interplay of selection and drift. More generally, the methods we present apply to any probability distribution {*μ*_*i*_} of initial mutant locations.

### Fixation probability

We define the *fixation probability* *ρ*_*G*_(*r*), for a mutant of fitness *r* on a graph *G*, as the probability that the state of all mutants is reached, from an initial state chosen according to the specified initialization scheme.

For the complete graph *K*_*N*_, representing a well-mixed population of size *N*, the fixation probability is [43]

$$\begin{array}{c} \rho_{K_{N}}\left(r\right)=\frac{1-r^{-1}}{1-r^{-N}}. \end{array}$$ (1)

More generally, Eq (1) holds for any graph that is *isothermal*, meaning that *T*_*i*_ = 1 for each vertex *i*. This is known as the Isothermal Theorem [1]. For Bd updating, the isothermal condition means that each vertex has the same probability of being replaced per time-step. For any isothermal graph *G* of size *N*, the fixation probability is given by [1]:

$$\begin{array}{c} \rho_{G}\left(r\right)=\frac{1-r^{-1}}{1-r^{-N}}. \end{array}$$ (2)

This result is valid for both uniform and temperature initialization, since the two schemes are equivalent on isothermal graphs.

Although calculating fixation probability is computationally intensive in general [6, 7, 30, 31], it simplifies when selection is weak; that is when mutations are nearly neutral (*r* ≈ 1). In this case, we may form the Taylor expansion

$$\begin{array}{c} \rho_{G}\left(1+\delta \right)=\rho^{\circ }+\delta \rho^{\prime }+O\left(\delta^{2}\right). \end{array}$$ (3)

**Fig 1:**
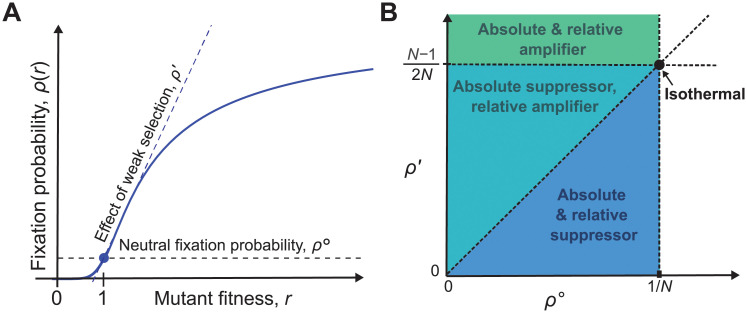
Classifying the effects of graph structure on weak selection. (**A**) The fixation probability *ρ*_*G*_(*r*), for a mutation of fitness *r* on a graph *G*, can be expanded under weak selection as $\rho_{G}\left(1+\delta \right)=\rho^{\circ }+\delta \rho^{\prime }+O\left(\delta^{2}\right)$. The zeroth-order coefficient, *ρ*^∘^, is the fixation probability of a neutral mutation, while the first-order coefficient, *ρ*′, determines the effect of weak selection. (**B**) The effects of graph structure on weak selection can be classified using *ρ*^∘^ and *ρ*′. All isothermal graphs (black dot) have *ρ*^∘^ = 1/*N* and *ρ*′ = (*N* − 1)/(2*N*). We say a graph is an *absolute amplifier* if *ρ*′ > (*N* − 1)/(2*N*), and an *absolute suppressor* if *ρ*′ < (*N* − 1)/(2*N*). We say a graph is a *relative amplifier* if *ρ*′/*ρ*^∘^ > (*N* − 1)/2 and a *relative suppressor* if *ρ*′/*ρ*^∘^ < (*N* − 1)/2. The three possible combinations for temperature initialization are shown in the colored regions.

The constant term, *ρ*^∘^ = *ρ*_*G*_(1), is the fixation probability of neutral mutations, while the linear term, $\rho^{\prime }=\frac{d}{dr}\rho_{G}{\left(r\right)|}_{r=1}$, represents the effect of weak selection (Fig 1A).

For isothermal graphs, Taylor expansion of Eq (2) gives

$$\begin{array}{c} \rho_{G}\left(1+\delta \right)=\frac{1}{N}+\delta \frac{N-1}{2N}+O\left(\delta^{2}\right). \end{array}$$ (4)

So *ρ*^∘^ = 1/*N* and *ρ*′ = (*N* − 1)/(2*N*) for all isothermal graphs.

Results of Allen et al. [14] imply that, for uniform initialization, *ρ*^∘^ = 1/*N* for all graphs. Thus spatial structure does not affect the rate of neutral drift when mutations appear uniformly. In contrast, for temperature initialization, Result 3 of Allen et al. [14] implies that *ρ*^∘^ ≤ 1/*N*, with equality if and only if the graph is isothermal.

### Amplifiers and suppressors

Our goal is to understand how spatial structure affects selection, neutral drift, and the relationship between the two. More specifically, we are interested whether a graph amplifies or suppresses selection, compared to the baseline of a well-mixed population. However, there are (at least) two distinct ways of making this comparison.

First, we can examine how the probability of fixation increases with fitness, as quantified by the first-order term, *ρ*′. It is intuitive that the rate of increase should be greater for amplifiers, and less for suppressors, relative to the well-mixed value of *ρ*′ = (*N* − 1)/(2*N*). This leads us to define a graph *G* as an *absolute amplifier of weak selection* if *ρ*′ > (*N* − 1)/(2*N*), and an *absolute suppressor of weak selection* if *ρ*′ < (*N* − 1)/(2*N*).

Second, to quantify the relationship between selection and neutral drift, we can compute the ratio of fixation probabilities for selected versus neutral mutations:

$$\begin{array}{c} \frac{\rho_{G}\left(1+\delta \right)}{\rho_{G}\left(1\right)}=\frac{\rho^{\circ }+\delta \rho^{\prime }}{\rho^{\circ }}+O\left(\delta^{2}\right)=1+\delta \frac{\rho^{\prime }}{\rho^{\circ }}+O\left(\delta^{2}\right). \end{array}$$ (5)

We see that the ratio of fixation probabilities, for weakly selected versus neutral mutations, is determined by *ρ*′/*ρ*^∘^. Consequently, *ρ*′/*ρ*^∘^ determines the ratio of beneficial to neutral mutations that accumulate over time. (This idea is reminiscent of the *dN*/*dS* or *K*_*a*_/*K*_*s*_ ratios that are used to study genetic sequence evolution [44].) For a well-mixed population (or any isothermal graph), *ρ*′/*ρ*^∘^ = (*N* − 1)/2. We therefore define a graph *G* to be a *relative amplifier of weak selection* if *ρ*′/*ρ*^∘^ > (*N* − 1)/2, and a *relative suppressor of weak selection* if *ρ*′/*ρ*^∘^ < (*N* − 1)/2.

For a family of graphs of unbounded size (*N* → ∞), we can compare lim_*N*→∞_ *ρ*′ to 1/2 to determine absolute amplifiers or suppressors, and lim_*N*→∞_ *ρ*′/(*Nρ*^∘^) to 1/2 to determine relative amplifiers or suppressors, in the large-population limit.

For uniform initialization, there is no distinction between the relative and absolute definitions, since *ρ*^∘^ = 1/*N* for all graphs. But for temperature initialization, the two notions are distinct. Moreover, since *ρ*^∘^ ≤ 1/*N* for all graphs [14], we have *ρ*′/*ρ*^∘^ ≥ *Nρ*′. This rules out the possibility that a graph can be simultaneously an absolute amplifier and a relative suppressor, leaving three possible classifications for non-isothermal graphs: (i) absolute and relative amplifier; (ii) absolute suppressor and relative amplifier; and (iii) absolute and relative suppressor. We illustrate these cases in Fig 1B.

## Results

Here we present our analytical and numerical results. Derivations are given in S1 Text

### Calculating fixation probability

To calculate the zeroth- and first-order coefficients, *ρ*^∘^ and *ρ*′ respectively, in the weak-selection expansion, Eq (3), we apply methods developed in previous works [14, 33, 45]. We address the zeroth-order (neutral drift) and first-order (weak selection) terms separately.

#### Neutral drift

To obtain the neutral fixation probability, *ρ*^∘^, we must first derive the fixation probability *π*_*i*_ of a neutral mutation arising at a particular vertex *i*. This probability *π*_*i*_ can also be understood as the reproductive value of vertex *i* [45, 46]. For Birth-death updating, the reproductive values *π*_*i*_ are the unique solution to the system of equations

$$\begin{array}{cc} T_{i}\pi_{i} & =\sum_{j\in G}p_{ij}\pi_{j}\;\text{for}\;\text{all}\;i\in G\text{,} \end{array}$$ (6a)

$$\sum_{i\in G}\pi_{i}=1.$$ (6b)

In the undirected case (*w*_*ij*_ = *w*_*ji*_), Eq (6) has an explicit solution in which reproductive value is inversely proportional to weighted degree: $\pi_{i}=w_{i}^{-1}/\tilde{W}$, where $\tilde{W}=\sum_{i\in G}w_{i}^{-1}$. In the directed case, Eq (6) appears not to have an explicit solution, but can be solved straightforwardly as a system of *N* + 1 linear equations (one redundant) in *N* variables.

The overall neutral fixation probability *ρ*^∘^, from an arbitrary probability distribution {*μ*_*i*_} of initial mutant locations, is then given by

$$\rho^{\circ }=\sum_{i\in G}\mu_{i}\pi_{i}.$$ (7)

For uniform initialization, we have $\rho^{\circ }=\frac{1}{N}\sum_{i\in G}\pi_{i}=1/N$ by Eq (6b), in agreement with previous work [14]. For temperature initialization (*μ*_*i*_ = *T*_*i*_/*N*) on an undirected graph (*w*_*ij*_ = *w*_*ji*_), substituting *μ*_*i*_ = *T*_*i*_/*N* and $\pi_{i}=w_{i}^{-1}/\tilde{W}$ gives

$$\begin{array}{c} \rho^{\circ }=\frac{1}{N\tilde{W}}\sum_{i\in G}\frac{T_{i}}{w_{i}}=\frac{1}{N\tilde{W}}\sum_{i,j\in G}\frac{w_{ij}}{w_{i}w_{j}}. \end{array}$$ (8)

#### Weak selection

To obtain the first-selection coefficient, *ρ*′, we turn to a method developed by McAvoy and Allen [33]. Consider an arbitrary initialization, characterized by a probability distribution {*μ*_*i*_} of initial mutant locations. Let *τ*_*ij*_ be the expected time, from initialization to fixation, that vertices *i* and *j* have different types. We prove in S1 Text that these *τ*_*ij*_ are uniquely determined by the recurrence relation

$$\begin{array}{c} \tau_{ij}=\{\begin{array}{cc} \frac{N\left(\mu_{i}+\mu_{j}\right)+\sum_{k\in G}\left(p_{ki}\tau_{kj}+p_{kj}\tau_{ki}\right)}{T_{i}+T_{j}} & i\neq j,\\ 0 & i=j. \end{array} \end{array}$$ (9)

In particular, for temperature initialization (*μ*_*i*_ = *T*_*i*_/*N*), we have

$$\begin{array}{c} \tau_{ij}=\{\begin{array}{cc} 1+\frac{\sum_{k\in G}\left(p_{ki}\tau_{kj}+p_{kj}\tau_{ki}\right)}{T_{i}+T_{j}} & i\neq j,\\ 0 & i=j. \end{array} \end{array}$$ (10)

In this case, *τ*_*ij*_ can be interpreted as the coalescence time from *i* and *j* [12, 36, 39–41, 47]; that is, the expected time for independent random walks from *i* and *j* to meet each other. These random walks can be taken to represent the ancestral lineages of the occupants of *i* and *j*, and *τ*_*ij*_ can be understood as the expected time to their most recent common ancestor [40, 41]. Eq (10) can be understood as follows: If *i* and *j* are the same vertex, the coalescence time is zero. Otherwise, we consider all the ways that either *i* or *j* could be replaced by the offspring of a neighbor, under neutral drift. Given that either *i* or *j* is replaced, the conditional probability that *i* is replaced by offspring of another vertex *k* is *p*_*ki*_/(*T*_*i*_ + *T*_*j*_), and the conditional probability that *j* is replaced by *k*’s offspring is *p*_*kj*_/(*T*_*i*_ + *T*_*j*_). When either of these events occurs, we increment time by one and consider the coalescence time of *k* and the vertex that was not replaced.

In the more general case of Eq (9), *τ*_*ij*_ can be thought of as a rescaled coalescence time, with the time spent at each vertex *k* ∈ *G* scaled proportionally to *μ*_*k*_. For uniform initialization (*μ*_*i*_ = 1/*N*) the recurrence becomes

$$\begin{array}{c} \tau_{ij}=\{\begin{array}{cc} \frac{2+\sum_{k\in G}\left(p_{ki}\tau_{kj}+p_{kj}\tau_{ki}\right)}{T_{i}+T_{j}} & i\neq j,\\ 0 & i=j. \end{array} \end{array}$$ (11)

For any initialization, we show in S1 Text that the weak-selection coefficient, *ρ*′, can be expressed in terms of the *τ*_*ij*_ as

$$\begin{array}{c} \rho^{\prime }=\frac{1}{2N\tilde{W}}\sum_{i,j}\frac{w_{ij}}{w_{i}w_{j}}\tau_{ij}. \end{array}$$ (12)

Together, Eqs (7), (9), and (12) allow for fixation probabilities to be computed under weak selection, for Bd updating on an arbitrary weighted graph, in polynomial time. In this way, one can efficiently determine the amplification and suppression properties of any given graph.

### Exhaustive analysis of small graphs

To explore the variety of possible effects of graph structure on fixation probabilities, we performed an exhaustive analysis of all connected simple graphs (unweighted, undirected, and with no self-loops) up to size 10, obtained from an online database [48]. For each graph, we calculated *ρ*′ and *ρ*^∘^ numerically, for both temperature and uniform initialization, by solving Eqs (10) or (11) and applying Eqs (8) and (12). We provide code to compute *ρ*′ and *ρ*^∘^ in Ref. [49].

#### Temperature initialization

**Fig 2:**
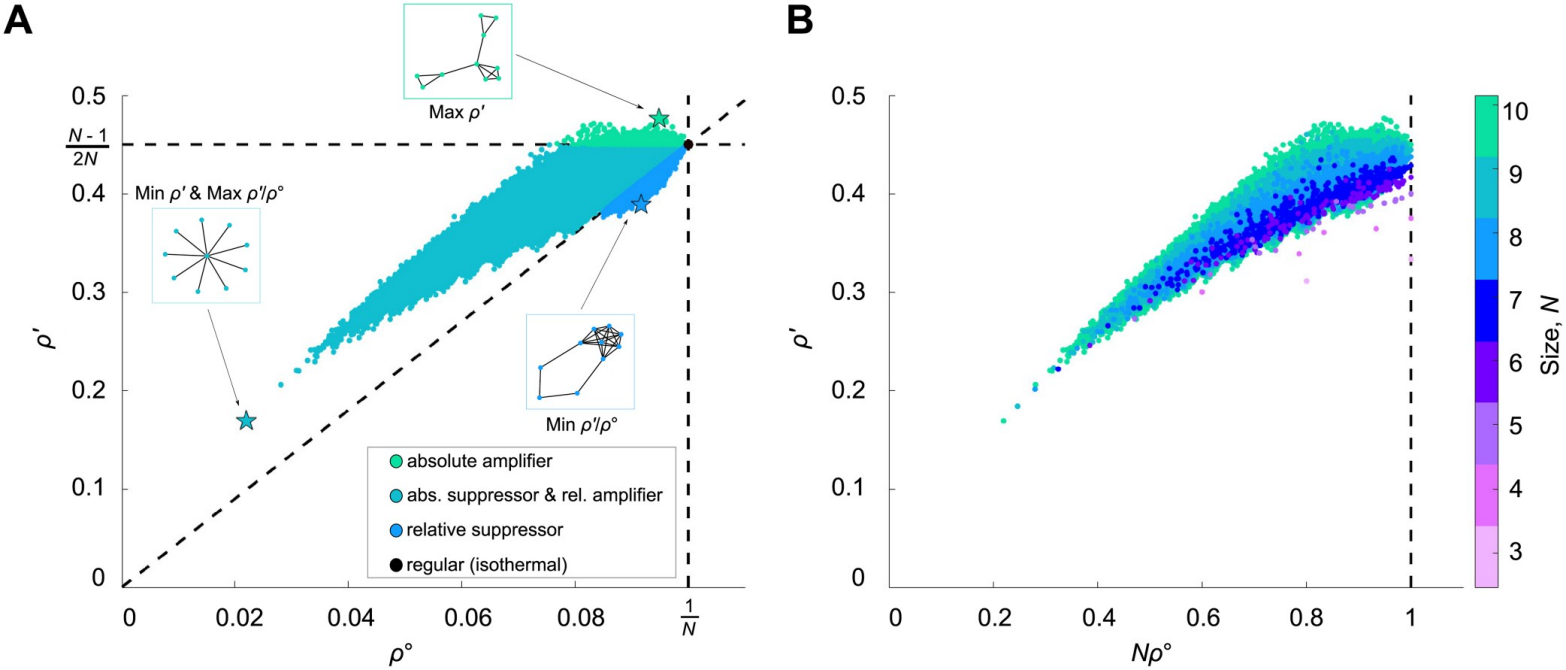
Exhaustive analysis of fixation probabilities under weak selection for small graphs. **(A)** The values of *ρ*^∘^ and *ρ*′ are plotted for all 11,716,571 connected unweighted graphs of size 10. Colors correspond to the classification of graphs as shown in Fig 1B. **(B)** Scatter plot of *ρ*′ versus *Nρ*^∘^ for all graphs up to size 10. Note that *Nρ*^∘^ ≤ 1 for all graphs, with equality if only if the graph is isothermal (or regular, in the context of unweighted graphs).

Our results for temperature initialization are summarized in Table 1 and Fig 2. We find that the vast majority of small unweighted graphs (99.6%, for *N* = 10) are relative amplifiers but absolute suppressors of weak selection. After that, for 8 ≤ *N* ≤ 10, absolute and relative suppressors are the next most abundant, followed by absolute and relative amplifiers, and finally isothermal graphs. (Note that an unweighted, undirected graph is isothermal if and only if it is regular.) Overall, there is a positive relationship between *ρ*^∘^ and *ρ*′, meaning that the graphs that slow the neutral molecular clock are also likely to suppress the (absolute) effects of weak selection.

**Table 1:** Classification of small graphs with temperature initialization.

Size, *N*	3	4	5	6	7	8	9	10
Absolute and relative suppressors	0	0	0	0	5	51	1,035	43,249
Absolute suppressors, relative amplifiers	1	4	19	106	838	11,006	259,776	11,671,038
Absolute and relative amplifiers	0	0	0	1	6	43	247	2,117
Isothermal (regular)	1	2	2	5	4	17	22	167
**Total**	2	6	21	112	853	11,117	261,080	11,716,571

**Fig 3:**
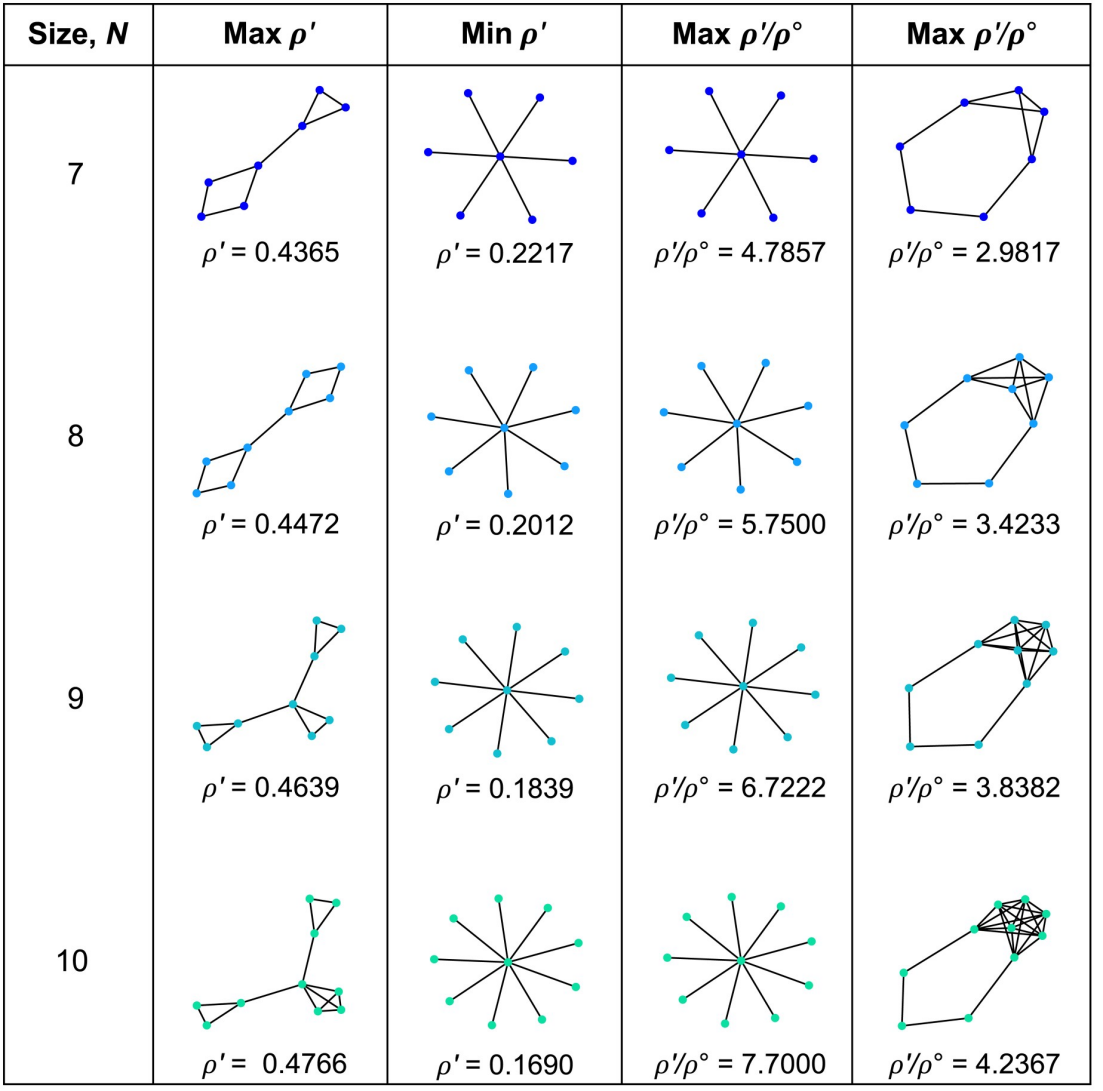
Small graphs with extreme effects for temperature initialization. The graphs with the largest or smallest values of *ρ*′ (which characterizes the likelihood of selected mutations to become fixed), and *ρ*′/*ρ*^∘^ (which quantifies the balance of selection versus drift) are shown for sizes 7 to 10. The Star graph minimizes *ρ*′ but maximizes *ρ*′/*ρ*^∘^ for these sizes. The largest *ρ*′ values arise for graphs with multiple components joined by single edges, while the smallest *ρ*′/*ρ*^∘^ ratios occur for Detour graphs.

We are particularly interested in the graphs that maximize or minimize *ρ*′ and *ρ*′/*ρ*^∘^ (Fig 3); these are the graphs with the most pronounced effects of spatial structure on natural selection. We find that the graphs that maximize *ρ*′ consist of several “islands” that are joined by single edges.

Interestingly, for all graphs up to size 10, the Star, *S*_*n*_, minimizes *ρ*′ and also maximizes the ratio *ρ*′/*ρ*^∘^. This suggests that in a star-structured population (with Bd updating and constant probability of mutation per birth), both neutral and advantageous mutations accrue much more slowly—but the ratio of advantageous to neutral is much larger—than in a well-mixed population of the same size.

The graphs that minimize the ratio *ρ*′/*ρ*^∘^ belong to a particular family, termed “Detours” by Möller et al. [10]. Detours are formed by starting with a complete graph and replacing one of the edges with a path of length ≥2.

#### Uniform initialization

**Fig 4:**
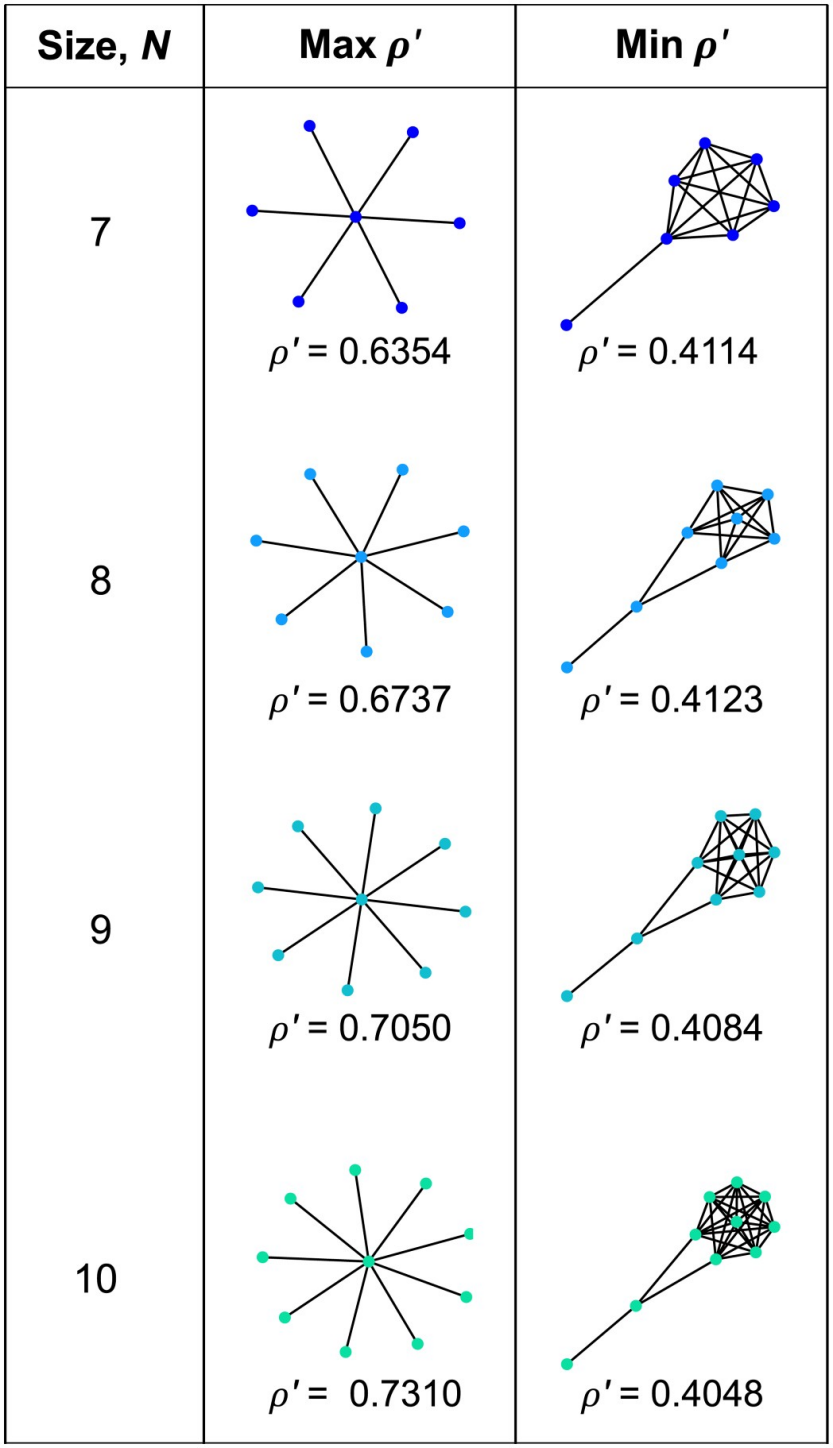
Small graphs with extreme effects for uniform initialization. The graphs with the largest or smallest values of *ρ*′ are shown for graphs of size 7 to 10. (Only *ρ*′ is shown because, for uniform initialization, *ρ*^∘^ = 1/*N* for every graph.) Star graphs have the largest *ρ*′, while the smallest *ρ*′ values appear for graphs with a highly connected component linked to a tail.

Our results for uniform initialization are presented in Table 2 and Fig 4. We find that the vast majority of small graphs (94%, in the case *N* = 10) are amplifiers of weak selection. This is consistent with previous numerical analyses [6, 9–11] which found that most small unweighted graphs are amplifiers for Bd with uniform initialization. In particular, the results for *N* = 6 and *N* = 7 agree with those from an exhaustive analysis performed by Cuesta et al. [9], who computed fixation probabilities for arbitrary mutant fitness *r*.

**Table 2:** Classification of small graphs with uniform initialization.

Size, *N*	3	4	5	6	7	8	9	10
Suppressors	0	0	1	7	55	671	14,890	659,784
Amplifiers	1	4	18	100	794	10,429	246,168	11,056,620
Isothermal (regular)	1	2	2	5	4	17	22	167
**Total**	2	6	21	112	853	11,117	261,080	11,716,571

The Star emerged as the strongest amplifier of weak selection, again consistent with previous results [10, 11, 50]. Stronger unweighted amplifiers of selection have been found for much larger populations [25], but not for population sizes *N* ≤ 100. Meanwhile, the strongest suppressors of weak selection have a well-connected portion joined by one or two edges to a tail—a structure we explore further below.

### Genetic algorithm

For graphs of size greater than 10, an exhaustive analysis is no longer feasible. To identify larger graphs that have extreme effects on fixation probability, we employed a genetic algorithm previously developed by Möller et al. [10]. The idea is to begin with a random ensemble of graphs, and select a subset with the largest or smallest values of a quantity of interest. These graphs are then “mated” with each other to produce an ensemble of “offspring” graphs, and the process is repeated. A formal description of the genetic algorithm and the parameters used is provided in S1 Text.

Based on our analysis of small graphs, we chose three targets to explore using the genetic algorithm: (i) maximal *ρ*′ for temperature initialization, (ii) minimal *ρ*′/*ρ*^∘^ for temperature initialization, and (iii) minimal *ρ*′ for uniform initialization. The other possible targets (minimal *ρ*′ and maximal *ρ*′/*ρ*^∘^ for temperature initialization, maximal *ρ*′ for uniform initialization) were optimized by the Star graph for all *N* ≤ 10, and so are presumably less interesting to explore.

**Fig 5:**
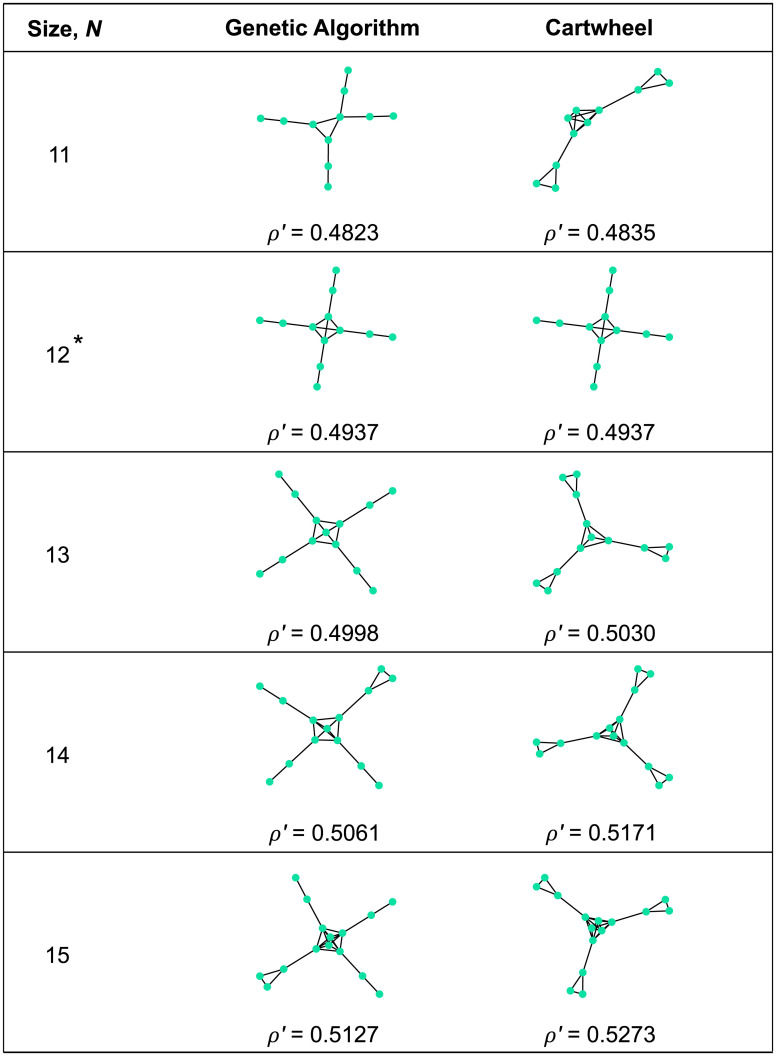
Discovering absolute amplifiers for temperature initialization. We used a genetic algorithm to discover graphs with large weak-selection effect *ρ*′. The resulting graphs (middle column) have a central “hub” joined by single links to outlying “islands”. To formalize this structure, we introduce a family of “Cartwheel” graphs *CW*_*n*,*m*,*h*_, consisting of a hub of size *h* and *n* islands of *m* vertices each (rightmost column). We find that the optimal Cartwheel graph has *ρ*′ exceeding that found by the genetic algorithm, except for *N* = 12 for which the same graph was identified by both methods. All graphs found by both methods are absolute amplifiers of weak selection, meaning *ρ*′ > (*N* − 1)/(2*N*).

**Fig 6:**
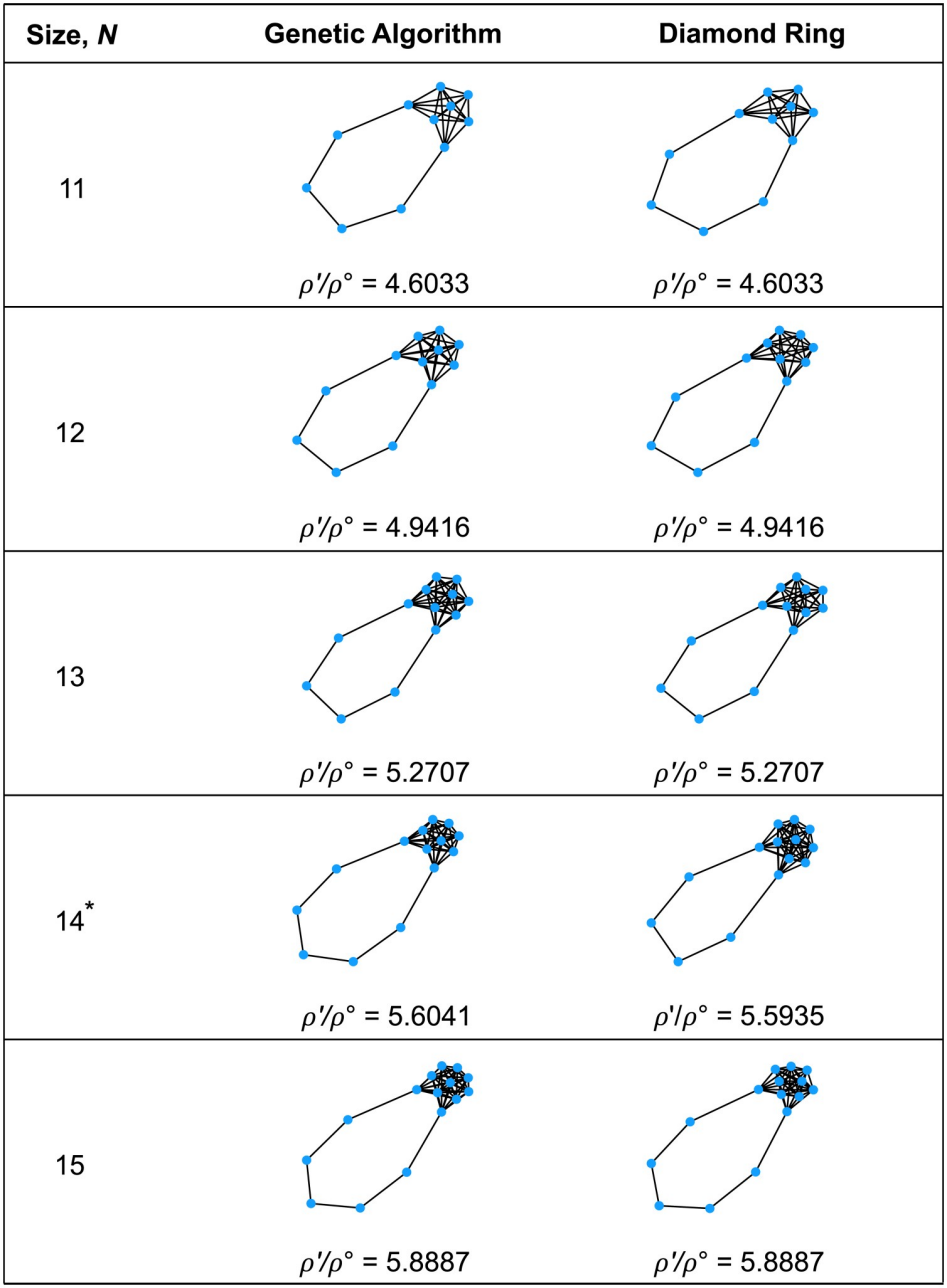
Discovering relative suppressors for temperature initialization. When seeking to minimize the ratio *ρ*′/*ρ*^∘^, the genetic algorithm, in all cases, found Detour graphs [10], consisting of a complete graph with one edge replaced by a path. For comparison, we calculated *ρ*′/*ρ*^∘^ for all Detour graphs of the given sizes. The results were identical to those of the genetic algorithm except in the case *N* = 14, for which the genetic algorithm found a Detour graph with a non-optimal number of ring vertices.

The results from the genetic algorithm extend and illuminate the patterns that were seen in the exhaustive analysis of smaller graphs. For temperature initialization, searching for large *ρ*′ (strong absolute amplifiers; Fig 5) leads to the emergence of a densely connected “hub”, joined by single edges to a number of “islands” consisting of two or more vertices each. Detour graphs, on the other hand, continue to appear when searching for small *ρ*′/*ρ*^∘^ (strong relative suppressors; Fig 6). We analyze both of these structures in-depth in later sections.

**Fig 7:**
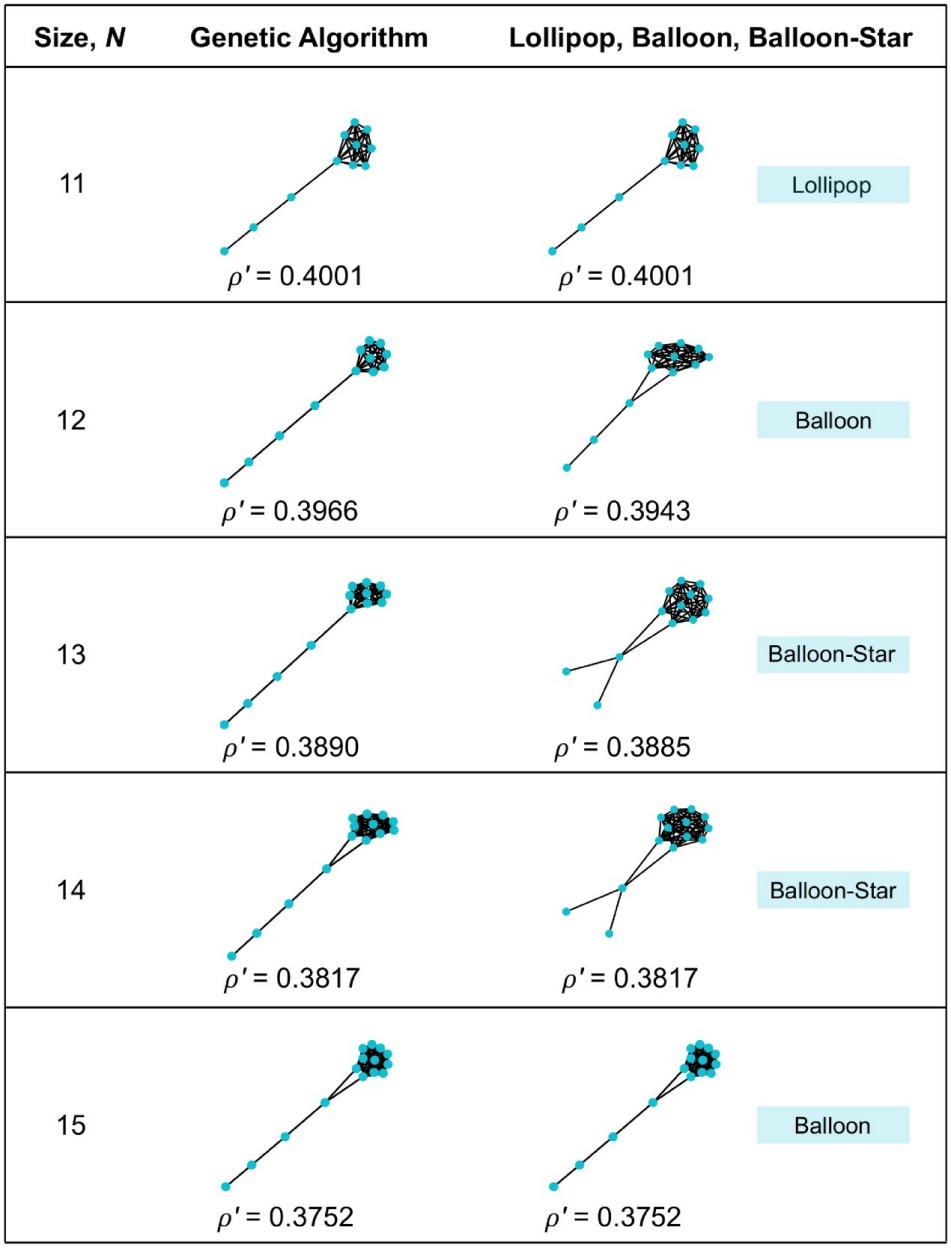
Discovering suppressors for uniform initialization. When seeking to minimize the ratio *ρ*′/*ρ*^∘^, the genetic algorithm produced structures consisting of a well-connected part and a tail. We compared these to Lollipop graphs (known for their random walk properties [51–53]), and two new families, which we call Balloons and Balloon-Stars. The minimal *ρ*′ from these families improved on the genetic algorithm results for *N* = 12, 13, 14 (albeit by less than 10^−4^ for *N* = 14), and matched the genetic algorithm results for *N* = 11 and *N* = 15.

For uniform initialization, searching for small *ρ*′ (strong suppressors; Fig 7) led to graphs with a well-connected part and a tail. The tails are longer than those found in the exhaustive search for *N* ≤ 10 (Fig 4). In some cases (*N* = 11, 12, 13, and also *N* = 7 in the exhaustive analysis) the tail connects at a single vertex to a clique, forming what is known as a Lollipop graph (also called a “standard kite” by Möller et al. [10]). Random walks on Lollipop graphs are known to have maximal hitting times [51], cover times [52], and commute times [53], so it is unsurprising that they have extreme effects on *ρ*′, which Eq (12) expresses in terms of the closely-related notion of coalescence times. However, in other cases (*N* = 14, 15, and *N* = 8, 9, 10 in the exhaustive search), the tail connects to two of the well-connected vertices, which are not connected to each other, forming what might be termed a “Balloon graph”. Additionally, other exploratory analyses (not shown) revealed some Balloon-like graphs for which the tail ends in a star; we call this a “Balloon-star”. We numerically computed the minimal *ρ*′ for all three of these families (Lollipop, Balloon, and Balloon-Star); the results are compared to the genetic algorithm results in Fig 7.

### Analysis of particular graph families

Here we analyze particular families of graphs that are found to have interesting properties. Derivations and proofs are given in S1 Text.

#### Star

**Fig 8:**
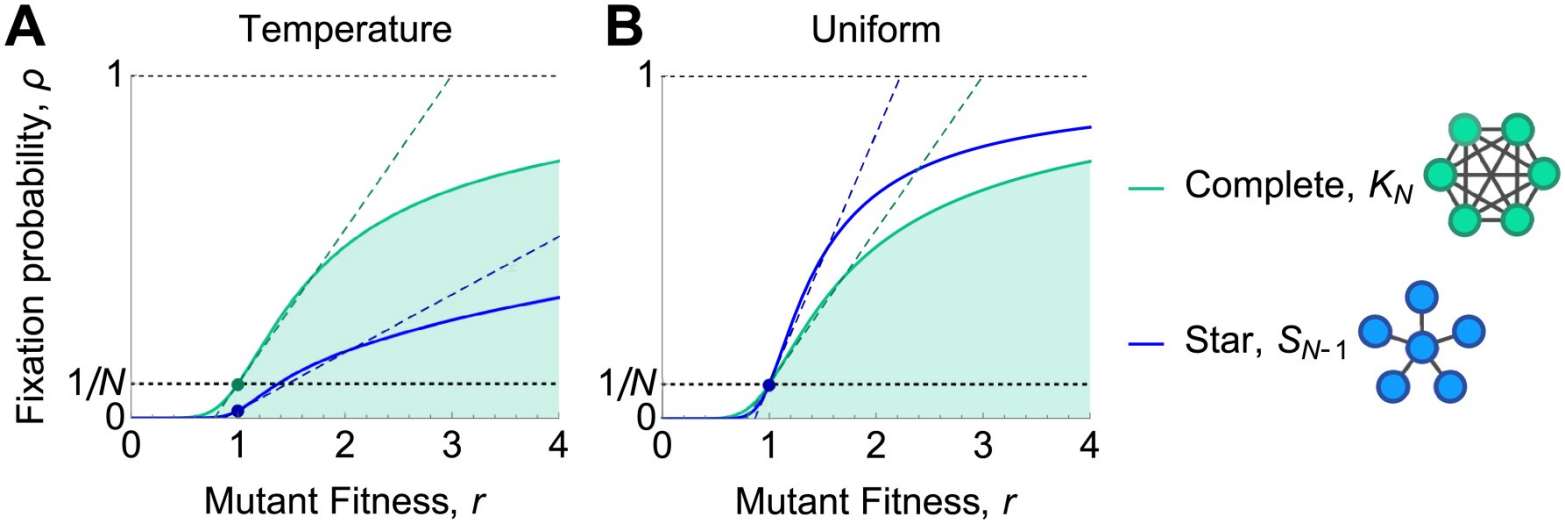
Star. Fixation probability, *ρ*(*r*) is plotted against mutant fitness *r*, for the Star and the complete graph. Dotted lines show the linear approximation *ρ*(1+ *δ*) ≈ *ρ*^∘^ + *δρ*′, accurate for weak selection (*δ* ≪ 1). **(A)** For temperature initialization, the Star is an absolute suppressor of weak selection, *ρ*′ < (*N* − 1)/(2*N*), but a relative amplifier, *ρ*′/*ρ*^∘^ > (*N* − 1)/2. **(B)** For uniform initialization, the Star is an amplifier of weak selection, *ρ*′ > (*N* − 1)/(2*N*). Note that *ρ*(1) = *ρ*^∘^ = 1/*N* for both graphs, as is true for any graph under uniform initialization.

The Star, *S*_*n*_ (Fig 8), consists of a single hub connected to each of *n* ≥ 2 leaves by an edge of weight 1. The Star was one of the first-identified amplifiers of selection for uniform initialization [1, 22]. For graphs of size *N* ≤ 10, our numerical investigation found Stars to be extremal in three different ways: the best absolute suppressors and relative amplifiers for temperature initialization, and the best amplifiers for uniform initialization.

For temperature initialization, solving Eqs (8), (10), and (12) yields

$$\rho^{\circ }=\frac{2n}{\left(n+1\right)\left(n^{2}+1\right)}$$ (13a)

$$\rho^{\prime }=\frac{n(2n^{2}-n+1)}{{\left(n+1\right)}^{2}(n^{2}+1)}.$$ (13b)

This agrees with Taylor expansion of previous results for the Star graph [22, 23, 54]. In S1 Text we prove that the Star is a relative amplifier and absolute suppressor for all population sizes. As the number of leaves *n* tends to infinity, the weak-selection coefficient *ρ*′ behaves as 2/*N* while the ratio *ρ*′/(*Nρ*^∘^) converges to one. Thus, for large sizes *N*, the Star is an arbitrarily strong absolute suppressor, and a relative amplifier by a factor of two (relative to the well-mixed population, for which lim_*N*→∞_ *ρ*′/(*Nρ*^∘^) = 1/2).

For uniform initialization, *ρ*^∘^ = 1/*N* (as for any graph), and Eqs (11), and (12) yield

$$\rho^{\prime }=\frac{n^{2}\left(n^{2}-n+2\right)}{{\left(n+1\right)}^{2}(n^{2}+1)}.$$ (14)

This again agrees with Taylor expansion of previous results for the Star [22, 23, 54]. We observe that lim_*n*→∞_ *ρ*′ = 1, meaning that the Star amplifies weak selection by a factor of two in this limit (relative to the well-mixed population, for which lim_*N*→∞_ *ρ*′ = 1/2). Although the Star is the strongest amplifier of weak selection for *N* ≤ 10, it is eventually (for sufficiently large *N*) surpassed by graphs in the Cartwheel family, as we describe below.

#### Complete bipartite graph

The complete bipartite graph, $K_{n_{A},n_{B}}$, is formed by partitioning the vertex set into two subsets, of respective sizes *n*_*A*_ and *n*_*B*_, and drawing an edge between each pair of vertices belonging to different subsets. No edges are drawn between vertices from the same subset. The Star, *S*_*n*_, is the special case *K*_1,*n*_, where one subset contains only the hub and the other subset contains all leaves. Tkadlec et al. [11] showed that complete bipartite graphs can amplify selection with only a relatively small increase in fixation time (relative to the well-mixed population).

In S1 Text we use our method to derive

$$\begin{array}{c} \rho^{\circ }=\frac{2n_{A}n_{B}}{\left(n_{A}+n_{B}\right)(n_{A}^{2}+n_{B}^{2})},\;\rho^{\prime }=\frac{n_{A}n_{B}(2\left(n_{A}^{2}+n_{B}^{2}\right)-\left(n_{A}+n_{B}\right))}{{\left(n_{A}+n_{B}\right)}^{2}(n_{A}^{2}+n_{B}^{2})}, \end{array}$$ (15)

for temperature initialization, and

$$\begin{array}{c} \rho^{\circ }=\frac{1}{N},\;\rho^{\prime }=\frac{{\left(n_{A}^{2}+n_{B}^{2}\right)}^{2}-\left(n_{A}^{3}+n_{B}^{3}\right)}{{\left(n_{A}+n_{B}\right)}^{2}(n_{A}^{2}+n_{B}^{2})}, \end{array}$$ (16)

for uniform initialization. These agree with previously-obtained results for the complete bipartite graph [4, 23], as well as with Eqs (13) and (14) for the Star. We prove in S1 Text that, for *n*_*A*_ ≠ *n*_*B*_, $K_{n_{A},n_{B}}$ is a relative amplifier but absolute suppressor of weak selection for temperature initialization, and an amplifier of weak selection for uniform initialization. For *n*_*A*_ = *n*_*B*_, $K_{n_{A},n_{B}}$ is isothermal, and so has the same fixation probabilities as a well-mixed population.

#### Cartwheel

**Fig 9:**
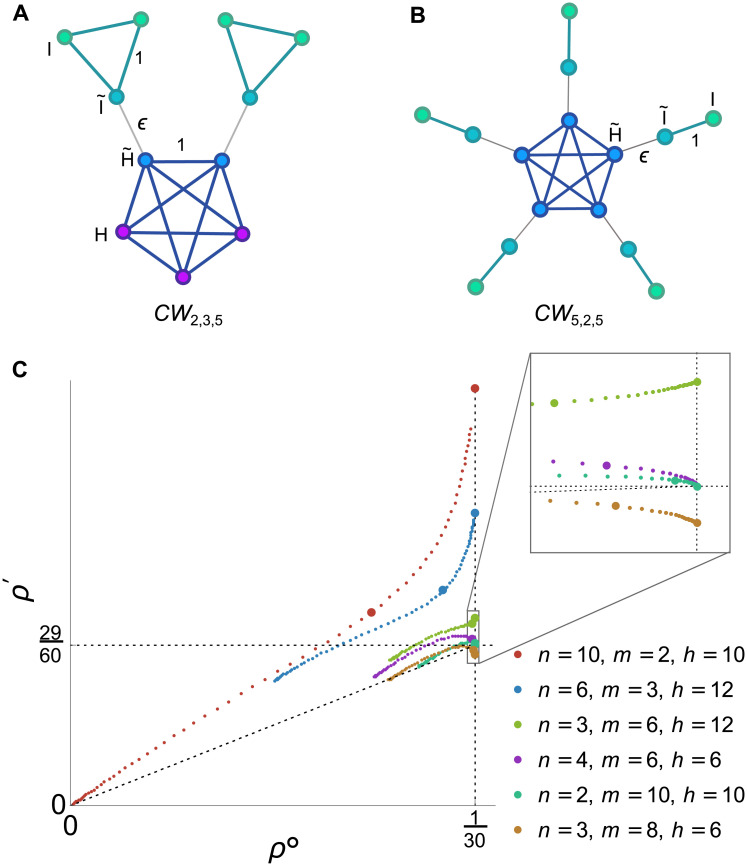
Cartwheel. **(A)** The Cartwheel graph *CW*_*n*,*m*,*h*_ contains *n* islands of *m* vertices each and *h* ≥ *n* hub vertices. Each island is connected to a distinct hub vertex by an edge of weight *ϵ*; vertices within the hub and within each island are joined by edges of weight 1. **(B)** The special case *n* = *h* and *m* = 2 has a “spider” structure; this graph has the largest *ρ*′ in the *ϵ* → 0 limit. **(C)** Plot of *ρ*^∘^ vs *ρ*′ for various Cartwheel graphs of size *N* = 30, with temperature initialization. Points are shown for each *ϵ* = 2^*k*^, where *k* varies from −5 to 9 in increments of 0.2. Larger points correspond to *ϵ* = 1 and the *ϵ* → 0 limit, as derived in Eq (19). Note that lim_*ϵ*→0_ *ρ*^∘^ = 1/*N* for all Cartwheel graphs. *CW*_10,2,10_ (the “spider” case) has by far the largest *ρ*′ in the *ϵ* → 0 limit; it also has the largest *ρ*/*ρ*^∘^, for all *ϵ*, among the graphs displayed. However, *CW*_6,3,12_ has the largest *ρ*′ for *ϵ* = 1 among Cartwheels of size 30. *CW*_4,6,6_ and *CW*_2,10,10_ both have *h* = *m* and therefore have the same fixation probability as a well-mixed population in the *ϵ* → 0 limit, according to Eq (18). *CW*_3,8,6_ has *h* < *m* and is therefore a suppressor of weak selection in the *ϵ* → 0 limit.

Both the exhaustive search and the genetic algorithm identified strong absolute amplifiers (large *ρ*′), for temperature initialization, consisting of a highly intraconnected “hub” joined by single edges to a number of “islands”. To formalize this pattern, we define a family of “Cartwheel” graphs *CW*_*n*,*m*,*h*_ (Fig 9A), consisting of an *h*-vertex hub and *n* islands of *m* vertices each. The hub and each island are cliques [55], meaning that within each subpopulation, each vertex is connected to each other. Each island is connected, by a single edge, to a distinct hub vertex. We generalized to a weighted graph by setting the hub-to-island edge weight to be a free parameter *ϵ* > 0; all other edge weights are 1. We consider only temperature initialization here, since this was the context in which Cartwheel graphs arose.

We have obtained closed-form expressions for *ρ*^∘^ and *ρ*′ for the Cartwheel using Mathematica. The formula for *ρ*^∘^ is given in S1 Text, while the formula for *ρ*′ is too lengthy to print. The behavior of *ρ*^∘^ and *ρ*′ as *ϵ* varies is illustrated in Fig 9C. As shown in Fig 5, there are unweighted Cartwheel graphs whose *ρ*′ values exceed those found by the genetic algorithm, suggesting that this family contains very strong absolute absolute amplifiers of weak selection. Table 3 shows the unweighted (*ϵ* = 1) Cartwheel graphs with the largest values of *ρ*′ for fixed sizes *N*. These *ρ*′ values eventually surpass 1, which is an upper bound for *ρ*′ values on the Star. We have not determined whether the *ρ*′ values for the unweighted Cartwheel are bounded, or whether they diverge to infinity as *N* increases.

**Table 3:** Unweighted Cartwheel graphs *CW*_*n*,*m*,*h*_ that maximize *ρ*′.

Size, *N*	*n*	*m*	*h*	*ρ*′
9	3	2	3	0.4622
10	2	3	4	0.4744
11	2	3	5	0.4835
12	4	2	4	0.4937
13	3	3	4	0.5030
14	3	3	5	0.5171
15	3	3	6	0.5273
20	4	3	8	0.5732
40	7	3	19	0.7082
60	10	3	30	0.7999
80	9	4	44	0.8827
100	11	4	56	0.9569
120	13	4	68	1.0183
140	12	5	80	1.0775

For arbitrary mutant fitness *r*, we derive (in S1 Text) the following closed-form expression for fixation probability in the *ϵ* → 0 limit:

$$\begin{array}{cc} \lim_{\epsilon \to 0}\rho_{CW_{n,m,h}}\left(r\right) & =\frac{\left(1-r^{-1})(r^{m+h}-1\right)}{\left(nm+h\right)(r^{m+h}-x^{n-1})}\\ & \times (\frac{\left(\frac{nm}{m-1}\right)}{r^{-m}(\frac{1-r^{-h}}{h-1})+(\frac{1-r^{-m}}{m-1})}+\frac{\left(\frac{h}{h-1}\right)}{\left(\frac{1-r^{-h}}{h-1})+r^{-h}(\frac{1-r^{-m}}{m-1}\right)}). \end{array}$$ (17)

**Fig 10:**
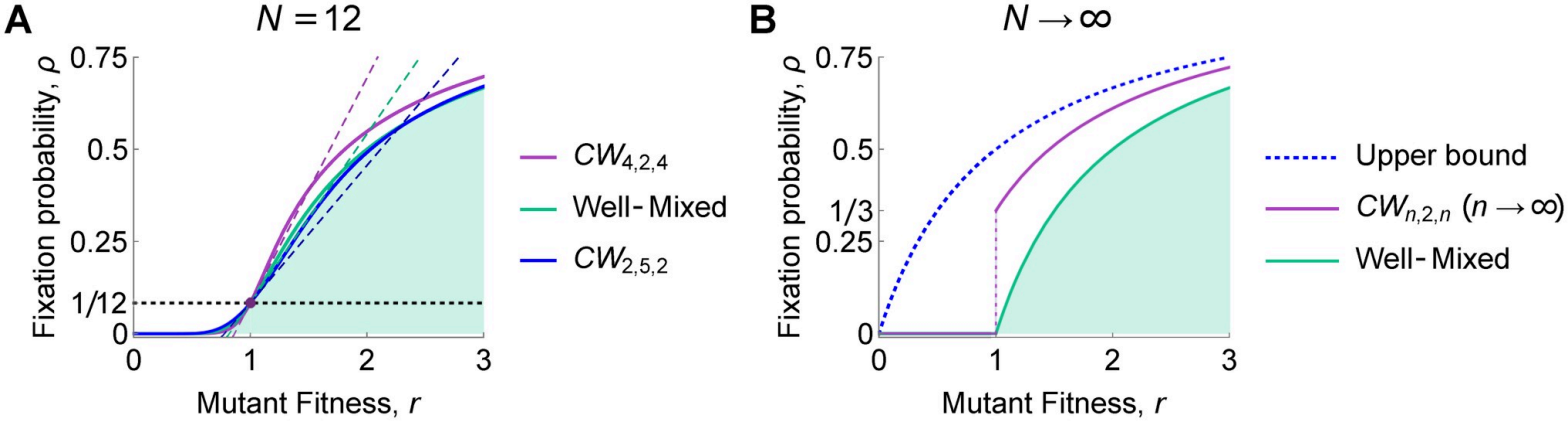
Nonweak selection on the Cartwheel graph. In the limit as the hub-to-island weight *ϵ* goes to zero, the fixation probability for arbitrary mutant fitness *r* is expressed in closed form by Eq (17). Temperature and uniform initialization are equivalent in this limit. Dashed lines show the weak-selection approximation, with slope *ρ*′ given by Eq (19). **(A)** Fixation probability is plotted against *r* for two Cartwheels of size 12. *CW*_4,2,4_ has *h* > *m* and is therefore an amplifier of weak selection. *CW*_2,5,2_ has *h* < *m* and is therefore a suppressor of weak selection (but appears to amplify selection for *r* > 2.4219). Cartwheels with *h* = *m* have the same fixation probability as the well-mixed population, for all *r*, in the *ϵ* → 0 limit. **(B)** In the limits *ϵ* → 0 and *n* = *h* → ∞, with *m* = 2, the fixation probability jumps discontinuously from 0 to 1/3 as *r* crosses 1; the expression for *r* > 1 is given in Eq (20). For comparison we also show the upper bound *ρ*(*r*) ≤ 1 − (*r* + 1)^−1^, derived by Pavlogiannis et al. [8], which applies to all weighted graphs with no self-loops under temperature initialization.

This result holds for both temperature and uniform initialization. In fact, the two initialization schemes become equivalent as *ϵ* → 0 since the temperature of each vertex converges to 1/*N*. (However, the Isothermal Theorem does not apply in this limit, since the graph is not exactly isothermal for any nonzero value of *ϵ*.) Several cases of Eq (17) are illustrated in Fig 10A.

If the hub is the same size as the islands, *h* = *m*, then Eq (17) reduces to

$$\begin{array}{c} \lim_{\epsilon \to 0}\rho_{CW_{n,m,m}}\left(r\right)=\frac{1-r^{-1}}{1-r^{-(mn+m)}}. \end{array}$$ (18)

This is exactly the fixation probability for a well-mixed population of the same size, *N* = *mn* + *m*. Thus for *h* = *m* and *ϵ* → 0, a mutant of arbitrary fitness *r* has the same fixation probability on the Cartwheel as in a well-mixed population.

Performing a Taylor expansion of Eq (17) around *r* = 1, we obtain *ρ*^∘^ = 1/*N* and

$$\begin{array}{c} \rho^{\prime }=\frac{N-1}{2N}+\frac{mnh\left(h-m\right)(m(n-2)(h-1)+h(m-1))}{2(h(m-1)+m(h-1))\left(mn+h\right)(mn(h-1)+h(m-1))}. \end{array}$$ (19)

The second term has the sign of *h* − *m*. It follows that the Cartwheel, in the *ϵ* → 0 limit, is an amplifier of weak selection for *h* > *m* and a suppressor of weak selection for *h* < *m*. These results hold in both the relative and absolute senses, for both temperature and uniform initialization.

The Cartwheel most strongly amplifies selection in the case *ϵ* → 0, *m* = 2, and *h* = *n* ≫ 1. This results in a “spider” structure, in which a large number of fully interconnected hub vertices are each joined (by an edge of vanishingly small weight) to a two-vertex “leg”. In this case, the fixation probability *ρ*(*r*) becomes (in the *ϵ* → 0 limit) discontinuous as a function of *r*: *ρ*(*r*) = 0 for disadvantageous mutations (*r* < 0), but for advantageous mutations (*r* > 0) we have

$$\begin{array}{c} \rho \left(r\right)=\frac{1-r^{-2}/3}{1+r^{-1}}. \end{array}$$ (20)

In particular, the fixation probability jumps from zero to 1/3 as *r* increases past 1. Any beneficial mutation has a fixation probability greater than one-third for such a structure, making this an especially powerful amplifier of natural selection.

We also show in S1 Text that the Cartwheel *CW*_*n*,2,*n*_, with *n* ≥ 7 and sufficiently small *ϵ*, is a stronger relative amplifier of weak selection than a Star of the same size.

#### Detour

**Fig 11:**
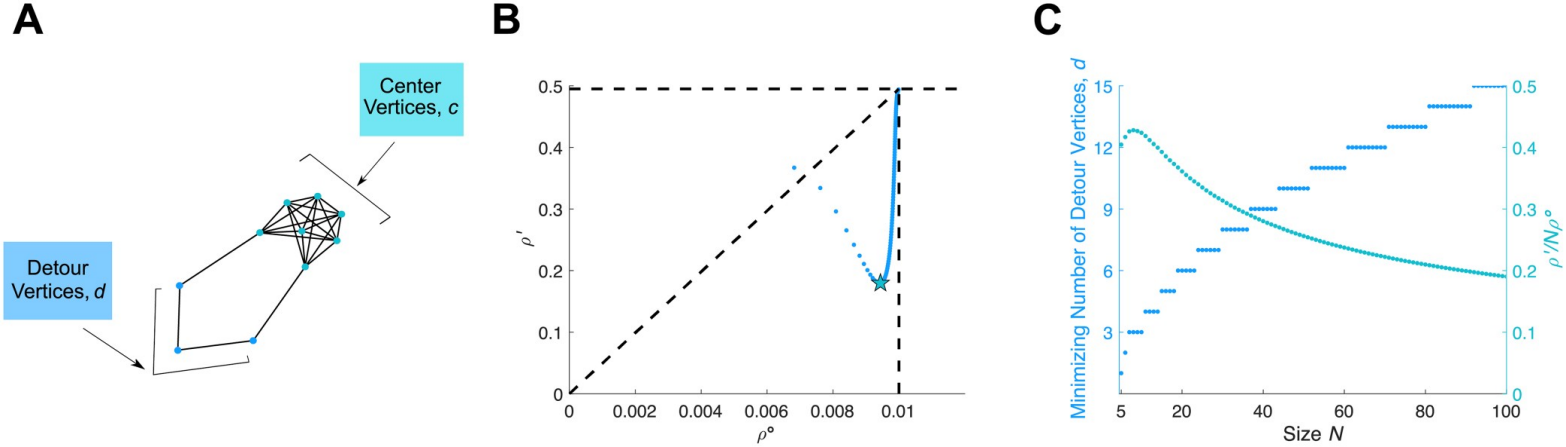
Detour. **(A)** The Detour graph *D*_*c*,*d*_ begins with a complete graph of size *c*, and replaces the edge between two vertices by a path with *d* interior vertices. **(B)** Values of *ρ*^∘^ and *ρ*′ are plotted as *d* varies, for *N* = 100. The Detour *D*_*c*,*d*_ is a relative suppressor of weak selection except for *d* = 1. The minimal ratio *ρ*′/*ρ*^∘^ is achieved for *d* = 15 (marked with a star). **(C)** The minimal *ρ*′/(*Nρ*^∘^) ratio, and the value of *d* achieving this ratio, is plotted for each *N*. Note that the minimizing *d* grows sub-linearly with *N*.

In the exhaustive analysis and genetic algorithm, Detour graphs [10] (Fig 11A) emerged as the strongest relative suppressors of weak selection (minimal *ρ*′/*ρ*^∘^) under temperature initialization. The Detour graph *D*_*c*,*d*_ is constructed by starting with a complete graph of size *c*, and replacing one edge with a path containing *d* vertices. The total size is *N* = *c* + *d*.

We have numerically determined the minimal *ρ*′/*ρ*^∘^ ratio for all sizes up to 100 (Fig 11B and 11C). As *N* increases beyond 8, the minimal *ρ*′/*ρ*^∘^ decreases, making these graphs increasingly strong relative suppressors. We also observe that the minimizing number of detour vertices *d* increases sublinearly with *N*.

#### Minimal absolute amplifier

**Fig 12:**
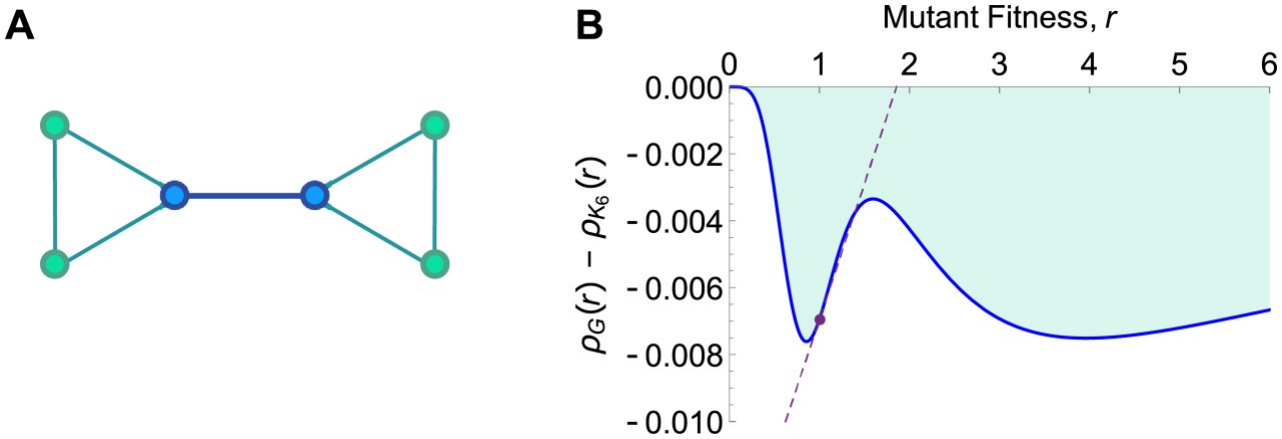
Minimal absolute amplifier. **(A)** This bowtie-shaped graph is the smallest absolute amplifier of weak selection under temperature initialization. **(B)** The difference in fixation probabilities between this graph and the complete graph *K*_6_ is plotted against mutant fitness, *r*. The dashed line shows the linear approximation to this difference at *r* = 1, computed using our weak-selection methods. Although this difference is increasing at *r* = 1 (because the graph is an absolute amplifier of weak selection), the difference is negative for all values of *r*. Thus this graph does not amplify selection in the usual sense of $\rho_{G}\left(r\right)>\rho_{K_{N}}\left(r\right)$ for all *r* > 1.

According to our small graph analysis in Table 1, there are no absolute amplifiers of weak selection of size ≤5, and only one of size 6. This graph of size 6 has a bowtie shape (Fig 12A) and is the minimal absolute amplifier of weak selection.

Due to its small size and symmetry, the fixation probability for this graph can be computed in closed form for arbitrary mutant fitness *r*. Employing an algorithm developed by Cuesta et al. [7, 9], adapted for temperature initialization, we obtained the fixation probability *ρ*_*G*_(*r*) as a ratio of 17th-degree polynomials (shown in S1 Text).

We find (Fig 12B) that, despite being an absolute amplifier of weak selection, this graph has fixation probability less than that of the complete graph *K*_6_ for all *r* > 0. This is possible because, although this graph increases the first-order term *ρ*′, relative to *K*_6_, it decreases the neutral term *ρ*^∘^, leading to a smaller overall fixation probability.

#### Fan

**Fig 13:**
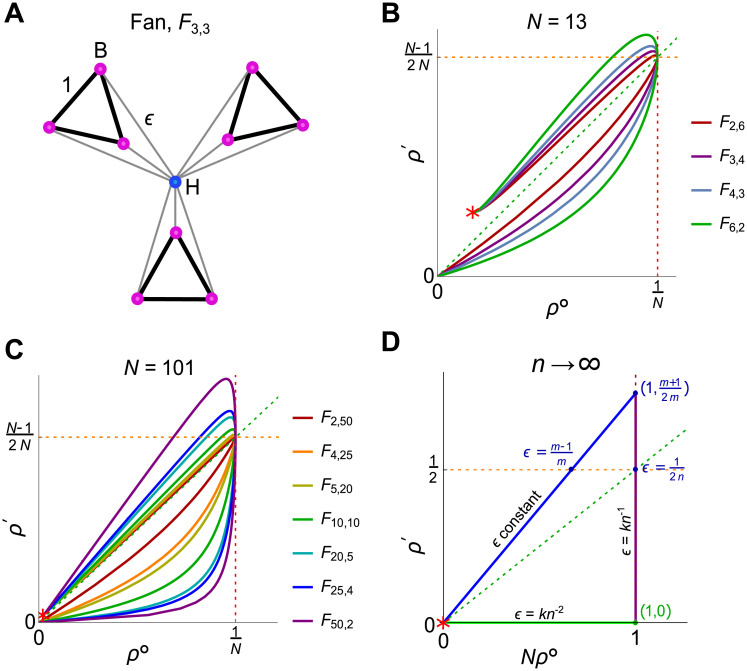
Fan. (A) The Fan *F*_*n*,*m*_ (or Windmill [56]) consists of one hub and *n* blades containing *m* vertices each. We consider a weighted version, with edge weights as shown. Pictured here is the case *n* = *m* = 3. **(B,C)** The neutral fixation probability *ρ*^∘^ and weak selection coefficient *ρ*′, plotted as *ϵ* varies from 0 to infinity, for all Fan graphs of sizes *N* = 13 and *N* = 101. As *ϵ* increases, the behavior changes from absolute and relative suppressor, to absolute and relative amplifier, to relative amplifier but absolute suppressor. For *ϵ* → ∞, the *ρ*^∘^ and *ρ*′ values approach those of the Star graph *S*_*nm*_ (marked by a red star). **(D)** As *n* → ∞, there are three regimes of behavior, depending whether *ϵ* is held constant (blue line) or scales as *n*^−1^ (purple line), or as *n*^−2^ (green line). The maximal *ρ*′ for fixed *m* is *ρ*′ = (*m* + 1)/(2*m*), achieved for *n* → ∞ with $\epsilon =\left(m-1\right)/\left(m\sqrt{n}\right)$.

Finally, we exhibit a graph family that displays all possible classifications of behavior as a particular edge weight is varied. The Fan, *F*_*n*,*m*_ [12] (or Windmill [56]), consists of a hub vertex attached to *n* ≥ 2 blades (Fig 13A). Each blade is a clique of *m* ≥ 2 vertices joined by edges of weight 1. The hub is connected to each blade vertex by an edge of weight *ϵ*. The total population size is *N* = *mn* + 1.

For the Fan *F*_*n*,*m*_ with temperature initialization, we find a neutral fixation probability of

$$\begin{array}{c} \rho^{\circ }=\frac{mn\epsilon (m-1+2\epsilon )(mn+2)}{\left(m-1+\epsilon \right)\left(mn+1\right)(m-1+\epsilon \left(m^{2}n^{2}+1\right))}. \end{array}$$ (21)

The first-order term is *ρ*′ = num/denom, with

$$\begin{array}{c} \text{num}=mn\epsilon (2{\left(m-1+\epsilon \right)}^{3}+2mn{\left(m-1+\epsilon \right)}^{2}\left(2m-1-\epsilon \right)\\ +2m^{2}n^{2}\left(m-1+\epsilon \right)\left(2\epsilon^{2}-\epsilon +m^{2}-m\right)\\ +2m^{4}n^{3}\epsilon \left(m+2\epsilon -1+nm^{2}-n\right))), \end{array}$$ (22a)

$$\begin{array}{c} \text{denom}=2\left(m-1+\epsilon \right)\left(mn+1\right)\left(m-1+\epsilon \left(m^{2}n^{2}+1\right)\right)({\left(m-1+\epsilon \right)}^{2}\\ +mn\left(1+\epsilon \right)\left(m-1+\epsilon \right)+m^{3}n^{2}\epsilon ). \end{array}$$ (22b)

We find that, as *ϵ* increases, *F*_*n*,*m*_ displays all three classifications of behavior. First, for 0 < *ϵ* < (*m* − 1)/(*mn* − 1), the Fan is both an absolute and relative suppressor. At *ϵ* = (*m* − 1)/(*mn* − 1), the graph is isothermal, and therefore has the same fixation probability as the well-mixed population. The graph is both an absolute and relative amplifier for (*m* − 1)/(*mn* − 1) < *ϵ* < ϵ*, where ϵ* is a particular cubic root for which *ρ*′ = (*N* − 1)/(2*N*). Then, for *ϵ* > ϵ*, the graph is a relative amplifier but absolute suppressor. As *ϵ* → ∞, the values of *ρ*^∘^ and *ρ*′ approach those of the star *S*_*nm*_, as given by Eq (13).

In the limit of many islands, *n* → ∞, the curve formed by *Nρ*^∘^ and *ρ*′ approaches a triangle (Fig 13D), the sides of which correspond to three scalings of *ϵ* with *n*: constant, inverse (*ϵ* = *kn*^−1^), and inverse square (*ϵ* = *kn*^−2^). The maximum value of *ρ*′ is achieved when *n* → ∞ with $\epsilon =\left(m-1\right)/\left(m\sqrt{n}\right)$; in this limit, *ρ*′ approaches (*m* + 1)/(2*m*).

For uniform initialization, *ρ*^∘^ = 1/*N* and *ρ*′ = num/denom, where

$$\begin{array}{c} \text{num}=m^{2}n^{2}\epsilon (m^{3}\left(m+1\right)n^{3}\epsilon +2m^{2}n^{2}\left(\epsilon -1\right)\epsilon \\ +mn\left(3m-2\epsilon +1\right)\left(m+\epsilon -1\right)+4{\left(m+\epsilon -1\right)}^{2}), \end{array}$$ (23a)

$$\begin{array}{c} \text{denom}=2\left(mn+1\right)(m^{2}n^{2}\epsilon +m+\epsilon -1)\\ \times (m^{3}n^{2}\epsilon +mn\left(\epsilon +1\right)\left(m+\epsilon -1\right)+{\left(m+\epsilon -1\right)}^{2}). \end{array}$$ (23b)

We show in S1 Text that the Fan with uniform initialization is a suppressor of weak selection for 0 < *ϵ* < (*m* − 1)/(*mn* − 1) and an amplifier of weak selection for *ϵ* > (*m* − 1)/(*mn* − 1). As *n* → ∞ with *m* and *ϵ* held constant, *ρ*′ converges to (*m* + 1)/(2*m*), matching the maximal limiting value of *ρ*′ that was found with temperature initialization.

## Discussion

The problem of identifying spatial structures that amplify or suppress selection has been actively investigated for the past decade and a half [1–13]. Our work introduces new methods for studying this problem in the weak selection regime. These methods allow for the effects of spatial structure on fixation probability (for *r* ≈ 1) to be computed in polynomial time, enabling analytical and computational investigations that would previously have been infeasible. However, this weak-selection approach does not tell us the behavior of *ρ*(*r*) away from *r* = 1, which may include complex phenomena such as multiple transitions between amplification and suppression [4, 9, 57]. We also have not explored the question of fixation time [10, 11, 26], which can significantly affect the overall rate of evolution [58].

Our work underscores the critical role that initialization schemes play in the determining how spatial structure affects selection [5, 11]. Different initialization schemes arise from different assumptions on how mutations occur. Uniform initialization, which is taken as the default in most previous works, corresponds to an assumption that mutation strikes all individuals equally regardless of age. Temperature initialization arises from instead considering a constant probability of mutation in each new offspring [14].

With temperature initialization comes new considerations, since the neutral fixation probability *ρ*^∘^ is affected in addition to the weak-selection coefficient *ρ*′. This leads to a distinction between relative versus absolute amplification and suppression. The absolute notions refer to the rate of increase fixation probability with respect to mutant fitness, while the relative notions refer to the expected ratio of beneficial to neutral mutations that fix over time. Of these, the relative notions are arguably more empirically relevant since they are directly related to observable genetic change.

A caveat with these definitions is that, for temperature initialization, absolute amplifiers of weak selection do not necessarily have larger fixation probability than the well-mixed population for any particular value of *r*. The minimal absolute amplifier (Fig 12) shows why: under temperature initialization, the neutral fixation probability *ρ*^∘^ is typically reduced from the well-mixed value, which may outweigh any increase in *ρ*′. On the other hand, it is certainly possible for a graph to have larger fixation probability than a well-mixed population under temperature initialization; the Cartwheel family provides examples of this.

Other initialization schemes aside from temperature and uniform may be considered. Since mutations can strike both existing organisms as well as new offspring, the relevant scheme for a given population may be a blend of uniform and temperature. Alternatively, for unicellular populations, mutations may occur in the parent cell upon reproduction; this would lead to initialization schemes that depend on the birth rate as well as the death rate. Fortunately, our method can be applied to any initialization scheme, using Eq (9) for coalescence times.

We have found that, for temperature initialization, the vast majority of small (unweighted, undirected) graphs are relative amplifiers but absolute suppressors of weak selection. This means that, for a spatially structured population satisfying the relevant modeling assumptions, one would typically see a greater ratio of beneficial-to-neutral mutations, but a slower overall rate of genetic change, compared to a well-mixed population. For uniform initialization, we find that the vast majority of graphs are amplifiers of weak selection, which accords with findings from other works using different methods [6, 10, 11].

Our findings reveal new families of graphs with interesting effects on selection, and shed new light on known families. In particular, our work has identified Cartwheel graphs (Fig 9) as strong absolute amplifiers for temperature initialization—with the “spider” case, *n* = *h* and *m* = 2, having especially interesting properties. In one limit, the fixation probability jumps discontinuously, from zero for all deleterious mutations, to more than one-third for all beneficial mutations (Fig 10). Stronger amplifiers for temperature initialization were found by Pavlogiannis et al. [8], but only with self-loops, meaning that new offspring can displace their own parents. Among graphs with no self-loops, Cartwheels appear to be the strongest family of amplifiers for temperature initialization that have been discovered thus far.

Detour graphs, in contrast, are shown to strongly suppress the *ρ*′/*ρ*^∘^ ratio for temperature initialization. This family of graphs was previously identified by Möller et al. [10] for their extreme properties under uniform initialization. In that context, Möller et al. found that Detours are powerful suppressors of selection for fitness values of *r* close to 1, but transition into amplifiers as *r* increases beyond a threshold value r* > 1. Overall, Detours appear to be a particularly interesting example for evolutionary graph theory. We have not come up with closed-form solutions for *ρ*^∘^ or *ρ*′ for Detours; doing so appears difficult although perhaps not impossible.

Our analysis of the Fan, meanwhile, highlights the determinative role that edge weight can play. By increasing the weight of a single type of edge, one can change the graph from a relative suppressor, to an absolute amplifier, to an absolute suppressor but relative amplifier. Interestingly, both *ρ*^∘^ and *ρ*′ are maximized for intermediate values of this edge weight. Attention to edge weight will therefore be crucial in connecting this theory to applications.

Although our general results apply to arbitrary (strongly connected) weighted digraphs, we focused our numerical exploration and examples on undirected graphs without self-loops. Extending this analysis to directed graphs would allow for many more possible structures, and may reveal new phenomena with regard to the amplification or suppression of selection.

## Conclusion

Our work and other recent contributions [5, 6, 9, 11–13, 27, 28] make clear that whether a population structure amplifies for suppresses selection depends not only on the graph, but also on the update rule, the initialization scheme, and the regime of fitness values being considered, and whether absolute or relative genetic change is of interest. This combinatorial explosion of modeling choices may seem daunting, but it also suggests an increased opportunity for applications of the theory. Different modeling choices will be relevant to animal, plant, and microbial populations, to somatic tissue [18–20], and to infectious diseases [15, 21]. Given recent advances in theory [8–13, 26, 33], the toolkit now exists to apply evolutionary graph theory in a wide range of biological settings.

## Supporting information

S1 Text Mathematical derivations and proofs. Contains derivations and proofs of our overall method, as well as mathematical analysis of particular graph families.
(PDF)
