# Supplementary material for: Fixation probabilities in graph-structured populations under weak selection
Source: PLoS Comput Biol. 2021 Feb 2;17(2):e1008695. doi: 10.1371/journal.pcbi.1008695 (PMC7880501; doi:10.1371/journal.pcbi.1008695)
Supplement: S1 Text — Contains derivations and proofs of our overall method, as well as mathematical analysis of particular graph families. (PDF) [file pcbi.1008695.s001.pdf]

S1 Text: Mathematical derivations and proofs

1 Model and Notation

This appendix contains derivations and proofs of the results in the main text. We begin with a technical review of our model and notation.

1.1 Graph

The population is structured as a weighted digraph, with the edge weight from vertex i to vertex j denoted w_{ij}. Self-loops, represented by $w_{ii} > 0$, are allowed. We require that the graph be *strongly connected*, meaning that there is a path of directed edges with nonzero weight from any given vertex to any other. Although our general results apply to directed graphs, we will pay special attention to the undirected case, which is when $w_{ij} = w_{ji}$ for each pair of vertices $i, j \in G$.

The *weighted out-degree* of a vertex $i \in G$ is defined as $w_i = \sum_{j \in G} w_{ij}$. The random walk step probability from i to j is $p_{ij} = w_{ij}/w_i$. The *temperature* of vertex i is defined to be $T_i = \sum_{j \in G} p_{ji}$. Note that $\sum_{i \in G} T_i = \sum_{i,j \in G} p_{ji} = N$.

1.2 Birth-death process

We let x_i denote the type occupying vertex $i \in G$: $x_i = 1$ if i is occupied by a mutant, and $x_i = 0$ if i is occupied by a resident. The types occupying each vertex are collected into a state vector $\mathbf{x} = (x_i)_{i \in G}$. We distinguish in particular the all-mutant state $\mathbf{1}$ and the all-resident state $\mathbf{0}$. The frequency of mutants in a given state $\mathbf{x}$ is denoted by $\bar{x} = \frac{1}{N} \sum_{i \in G} x_i$.

Mutants have fitness $r = 1 + \delta$, while residents have fitness 1. So overall, the fitness of the occupant of vertex i can be written as $1 + \delta x_i$.

According to the Birth-death (Bd) process, first an individual is chosen to reproduce proportionally to fitness, and then it displaces a neighbor chosen proportionally to edge weight. The probability that the offspring of vertex i replaces the occupant of j in state $\mathbf{x}$ in a single time-step is therefore

$$\begin{aligned} e_{ij}(\mathbf{x}) &= \left(\frac{1+\delta x_i}{N+\delta\sum_{k\in G} x_k}\right) p_{ij} \\ &= \left(\frac{1+\delta x_i}{1+\delta\bar{x}}\right)\frac{p_{ij}}{N}. \end{aligned} \tag{1}$$

1.3 Initialization

We consider an initial state in which a single vertex is occupied by a mutant, and all other vertices are occupied by residents. The vertex containing the initial mutant is chosen from a probability distribution $\mu = \{\mu_i\}_{i\in G}$; we call this distribution the *initialization*.

We focus on two cases: *uniform initialization*, in which the mutant is equally likely to appear at each vertex, $\mu_i = 1/N$ for each $i \in G$, and *temperature initialization*, in which mutants appear proportionally to temperature, $\mu_i = T_i/N$.

2 Weak-selection expansion of fixation probability

Here we derive the formulas for ρ° and ρ', in the Taylor expansion $\rho(1+\delta) = \rho^\circ + \delta\rho' + \mathcal{O}(\delta^2)$, that are given in the main text.

2.1 Neutral drift

We begin with the netural drift term ρ°. Neutral drift is the case $r = 1$ (equivalently, $\delta = 0$), meaning that mutants and residents have the same fitness. Quantities that pertain to neutral drift are identified with a $^\circ$.

Under neutral drift, Eq. (1), for the probability that the offspring of i replaces the occupant of j, reduces to

$$e^\circ_{ij} = \frac{p_{ij}}{N} = \frac{w_{ij}}{Nw_i}. \tag{2}$$

Importantly, the neutral replacement probabilities e°_{ij} are independent of the population state $\mathbf{x}$. From these replacement probabilities, we can calculate the neutral birth and death rate of vertex i,

$$b^{\circ}_i = \sum_{j \in G} e^{\circ}_{ij} = \frac{1}{N}, \tag{3}$$

and

$$d^{\circ}_i = \sum_{j \in G} e^{\circ}_{ji} = \frac{1}{N} \sum_{j \in G} p_{ji} = \frac{1}{N} \sum_{j \in G} \frac{w_{ji}}{w_j} = \frac{T_i}{N}. \tag{4}$$

We now turn to fixation probability. Let π_i denote the probability, under neutral drift, that a mutant type arising at vertex i will reach fixation (i.e. that the process reaches the all-mutant state $\mathbf{1}$ from an initial state with a mutant in vertex i and residents elsewhere). We refer to π_i as the *reproductive value* (RV) of vertex i [1, 2, 3]. These reproductive values π_i are determined by the following system of equations [4, 5].

$$d^{\circ}_i \pi_i = \sum_{j \in G} e^{\circ}_{ij} \pi_j \tag{5a}$$

$$\sum_{i \in G} \pi_i = 1. \tag{5b}$$

For Bd updating, Eq. (5a) becomes

$$\sum_{j \in G} p_{ji} \pi_i = \sum_{j \in G} p_{ij} \pi_j, \tag{6}$$

which is equivalent to Eq. (6a) of the main text.

In the case of an undirected graph ($w_{ji} = w_{ij}$ for all $i, j \in G$), we can solve Eq. (5) explicitly to obtain

$$\pi_i = \frac{w_i^{-1}}{\tilde{W}}, \qquad \text{where } \tilde{W} = \sum_{j \in G} w_j^{-1}. \tag{7}$$

In this case, each vertex has reproductive value inversely proportional to its weighted degree. For directed graphs, there is no explicit solution, but Eq. (5) can be solved using standard methods for systems of linear equations.

We now consider an arbitrary initialization $\mu = \{\mu_i\}_{i \in G}$. The overall neutral fixation probability, ρ°, is then given by

$$\rho^\circ = \sum_{i \in G} \mu_i \pi_i. \tag{8}$$

For uniform initialization, $\mu_i = 1/N$ for each i, which implies that $\rho^\circ = \frac{1}{N} \sum_{i \in G} \pi_i = \frac{1}{N}$, independently of the graph structure. For temperature initialization, we have $\mu_i = d_i^\circ = T_i/N$, leading to

$$\rho^\circ = \sum_{i \in G} d_i^\circ \pi_i = \frac{1}{N} \sum_{i \in G} T_i \pi_i. \tag{9}$$

Allen et al. [4] proved that, if the neutral birth rate b_i° is uniform over vertices (which is always true for Birth-death updating), then $\rho^\circ \leq 1/N$. Equality occurs only when d_i° is also uniform over vertices, which for Bd updating occurs only when the graph is isothermal.

In the case of an undirected graph, substituting Eq. (7) into Eq. (9) gives the explicit formula

$$\rho^\circ = \frac{1}{N\tilde{W}} \sum_{i,j \in G} \frac{w_{ij}}{w_i w_j}. \tag{10}$$

2.2 Weak selection

To compute the first-order term ρ', which quantifies the effects of weak selection, we employ a method developed by McAvoy and Allen [6].

2.2.1 Change due to selection

We begin by defining the RV-weighted frequency of mutants:

$$\hat{x} = \sum_{i \in G} \pi_i x_i. \tag{11}$$

Let $\hat{\Delta}_{\text{sel}}(\mathbf{x})$ denote the expected change in the RV-weighted frequency $\hat{x}$ from a given state $\mathbf{x}$, which can be expressed using Eq. (1) as

$$\begin{aligned}\hat{\Delta}_{\text{sel}}(\mathbf{x}) &= \sum_{i,j \in G} e_{ij}(\mathbf{x})\ (x_i - x_j)\pi_j \\ &= \frac{1}{N} \sum_{i,j \in G} \left(\frac{1 + \delta x_i}{1 + \delta \bar{x}} \right) p_{ij}(x_i - x_j)\pi_j. \end{aligned} \tag{12}$$

For the neutral case, setting $\delta = 0$ yields

$$\begin{aligned}
\hat{\Delta}^{\circ}_{\text{sel}}(\mathbf{x}) &= \frac{1}{N} \sum_{i,j \in G} p_{ij}(x_i - x_j)\pi_j \\
&= \frac{1}{N} \sum_{i \in G} x_i \left(\sum_{j \in G} p_{ij}\pi_j - \sum_{j \in G} p_{ji}\pi_i \right) \\
&= 0,
\end{aligned} \tag{13}$$

by Eq. (5a). Turning to weak selection, we denote by $\hat{\Delta}'_{\text{sel}}(\mathbf{x})$ the derivative of $\hat{\Delta}_{\text{sel}}(\mathbf{x})$ with respect to δ at $\delta = 0$. Taking the derivative of Eq. (12) and applying Eq. (13) and the fact that $x_i^2 = x_i$ for each i, we obtain

$$\begin{aligned}
\hat{\Delta}'_{\text{sel}}(\mathbf{x}) &= \left.\frac{d}{d\delta}\right|_{\delta=0} \hat{\Delta}_{\text{sel}}(\mathbf{x}) \\
&= \frac{1}{N} \sum_{i,j \in G} (x_i - \bar{x})\, p_{ij}(x_i - x_j)\pi_j \\
&= \frac{1}{N} \sum_{i,j \in G} x_i p_{ij}(x_i - x_j)\pi_j - \frac{\bar{x}}{N} \sum_{i,j} p_{ij}(x_i - x_j)\pi_j \\
&= \frac{1}{N} \sum_{i,j \in G} x_i p_{ij}(x_i - x_j)\pi_j \\
&= \frac{1}{N} \sum_{i,j \in G} p_{ij} x_i \pi_j - \frac{1}{N} \sum_{i,j \in G} p_{ij} x_i x_j \pi_j.
\end{aligned} \tag{14}$$

Using Eq. (5a), we observe that the sum in first term can be rewritten as follows:

$$\begin{aligned}
\sum_{i,j \in G} p_{ij} x_i \pi_j &= \sum_{i \in G} x_i \sum_{j \in G} p_{ij}\pi_j \\
&= \sum_{i \in G} x_i \sum_{j \in G} p_{ji}\pi_i \\
&= \sum_{i,j \in G} p_{ji} x_i \pi_i \\
&= \sum_{i,j \in G} p_{ij} x_j \pi_j.
\end{aligned}$$

Thus, overall, we can rewrite Eq. (14) as

$$\begin{aligned}\hat{\Delta}'_{\text{sel}}(\mathbf{x}) &= \frac{1}{2N}\sum_{i,j\in G} p_{ij}x_i\pi_j + \frac{1}{2N}\sum_{i,j\in G} p_{ij}x_j\pi_j - \frac{1}{N}\sum_{i,j\in G} p_{ij}x_ix_j\pi_j \\ &= \frac{1}{2N}\sum_{i,j} p_{ij}\pi_j\big(x_i(1-x_j)+x_j(1-x_i)\big) \\ &= \frac{1}{2N}\sum_{i,j} p_{ij}\pi_j\,\chi_{x_i\neq x_j}(\mathbf{x}). \end{aligned} \tag{15}$$

Above, $\chi_{x_i\neq x_j}(\mathbf{x})$ is an indicator function, equal to 1 if $x_i \neq x_j$ in state $\mathbf{x}$ and 0 otherwise. We observe that $\hat{\Delta}'_{\text{sel}}$ is greatest when neighboring vertices have different types.

2.2.2 Fixation probability

Let the random vector $\mathbf{X}(t) = (X_i(t))_{i\in G}$ represent the state of the process at time t. Let $\hat{X}(t) = \sum_{i\in G}\pi_i X_i$ represent the RV-weighted frequency at time t. Let $\mathbb{E}_{\mu,r}$ denote an expectation of a random variable, given that the mutant fitness is r and the vertex containing the intial mutation is sampled from the probability distribution $\mu = \{\mu_i\}_{i\in G}$.

Noting that the RV-weighted frequency $\hat{x}$ is 1 for $\mathbf{x} = \mathbf{1}$ and 0 for $\mathbf{x} = \mathbf{0}$, we have

$$\begin{aligned}\rho(r) &= \lim_{t\to\infty}\mathbb{E}_{\mu,r}[\hat{X}(t)] \\ &= \mathbb{E}_{\mu,r}[\hat{X}(0)] + \sum_{t=0}^{\infty}\mathbb{E}_{\mu,r}[\hat{X}(t+1)-\hat{X}(t)] \\ &= \rho^\circ + \sum_{t=0}^{\infty}\mathbb{E}_{\mu,r}[\hat{\Delta}_{\text{sel}}(\mathbf{X}(t))],\end{aligned} \tag{16}$$

using Eq. (8).

We now turn to weak selection by setting $r = 1+\delta$ with $|\delta| \ll 1$. We let $\mathbb{E}^\circ_\mu = \mathbb{E}_{\mu,1}$ denote expectations for the neutral case ($r = 1$, or equivalently, $\delta = 0$). Since $\hat{\Delta}^\circ_{\text{sel}}(\mathbf{x}) = 0$ for each state $\mathbf{x}$, we have

$$\mathbb{E}_{\mu,1+\delta}[\hat{\Delta}_{\text{sel}}(\mathbf{X}(t))] = \delta\,\mathbb{E}^\circ_\mu[\hat{\Delta}'_{\text{sel}}(\mathbf{X}(t))] + \mathcal{O}(\delta^2). \tag{17}$$

Let us introduce the operator $\langle\ \rangle_\mu^\circ$ on state functions $\phi(\mathbf{x})$ by

$$\langle\phi\rangle_\mu^\circ = \sum_{t=0}^{\infty} \mathbb{E}_\mu^\circ\left[\phi(\mathbf{X}(t))\right]. \tag{18}$$

McAvoy and Allen [6, Corollary 1] prove that sum on the right-hand side converges absolutely for any function $\phi(\mathbf{x})$ satisfying $\phi(\mathbf{1}) = \phi(\mathbf{0}) = 0$.

Substituting Eq. (17) into (16) and interchanging sums and derivatives (which is justified by uniform convergence), we obtain the expansion

$$\rho(1+\delta) = \rho^\circ + \delta\left\langle \hat{\Delta}'_{\text{sel}}\right\rangle_\mu^\circ + O\left(\delta^2\right). \tag{19}$$

The validity of Eq. (19) is proven formally by McAvoy and Allen [6, Theorem 1]. Combining with Eq. (15) have the following expression for the weak-selection (first-order) coefficient ρ':

$$\rho' = \left\langle \hat{\Delta}'_{\text{sel}}\right\rangle_\mu^\circ = \frac{1}{2N}\sum_{i,j\in G} p_{ij}\pi_j \left\langle \chi_{x_i\neq x_j}\right\rangle_\mu^\circ. \tag{20}$$

Defining $\tau_{ij} = \left\langle \chi_{x_i\neq x_j}\right\rangle_\mu^\circ$, we have

$$\rho' = \frac{1}{2N}\sum_{i,j\in G} p_{ij}\pi_j\tau_{ij}. \tag{21}$$

In the undirected case, substituting $p_{ij} = w_{ij}/w_i$ and $\pi_j = w_j/\tilde{W}$, as per Eq. (7), gives

$$\rho' = \frac{1}{2N\tilde{W}}\sum_{i,j\in G} \frac{w_{ij}\tau_{ij}}{w_i w_j}. \tag{22}$$

2.2.3 Recurrence relation for coalescence lengths

In the previous section we defined the coalescence lengths τ_{ij} by

$$\tau_{ij} = \left\langle \chi_{x_i\neq x_j}\right\rangle_\mu^\circ = \sum_{t=0}^{\infty} \mathbb{P}_\mu^\circ\left[X_i(t)\neq X_j(t)\right], \tag{23}$$

It remains to derive the recurrence relation for τ_{ij}. It is clear from Eq. (23) that $\tau_{ii} = 0$ and $\tau_{ij} = \tau_{ji}$ for each $i, j \in G$. For $i \neq j$, we expand Eq. (23) as

$$\tau_{ij} = \mathbb{P}_\mu^\circ\left[X_i(0)\neq X_j(0)\right] + \sum_{t=0}^{\infty} \mathbb{P}_\mu^\circ\left[X_i(t+1)\neq X_j(t+1)\right]. \tag{24}$$

We note that $X_i(0) \neq X_j(0)$ only if the initial mutant appears at either i or j, so the first term on the right-hand side reduces to

$$\mathbb{P}^\circ_\mu \left[X_i(0) \neq X_j(0)\right] = \mu_i + \mu_j. \tag{25}$$

For the second term, considering all the ways the occupants of i or j could be replaced, under neutral drift, from time t to time $t+1$, we have

$$\begin{aligned}
&\mathbb{P}^\circ_\mu \left[X_i(t+1) \neq X_j(t+1)\right] \\
&\qquad = \sum_{k \in G} \left(e^\circ_{ki}\, \mathbb{P}^\circ_\mu \left[X_k(t) \neq X_j(t)\right] + e^\circ_{kj}\, \mathbb{P}^\circ_\mu \left[X_i(t) \neq X_k(t)\right] \right) \\
&\qquad\qquad + \left(1 - \sum_{k \in G} (e^\circ_{ki} + e^\circ_{kj}) \right) \mathbb{P}^\circ_\mu \left[X_i(t) \neq X_j(t)\right]. \qquad (26)
\end{aligned}$$

Substituting Eqs. (26) and (25) into (24), and invoking Eq. (23), $e^\circ_{ij} = p_{ij}/N$, and $T_i = \sum_{k \in G} p_{ki}$, we have

$$\tau_{ij} = \mu_i + \mu_j + \frac{1}{N} \sum_{k \in G} \left(p_{ki}\tau_{kj} + p_{kj}\tau_{ik}\right) + \left(1 - \frac{T_i + T_j}{N}\right) \tau_{ij}. \tag{27}$$

Solving for τ_{ij}, and combining with the $i = j$ case, we have the recurrence relation

$$\tau_{ij} = \begin{cases} \dfrac{N(\mu_i + \mu_j) + \sum_{k \in G} \left(p_{ki}\tau_{kj} + p_{kj}\tau_{ik}\right)}{T_i + T_j} & i \neq j \\ 0 & i = j. \end{cases} \tag{28}$$

In particular, for temperature initialization, $\mu_i = T_i/N$, we have

$$\tau_{ij} = \begin{cases} 1 + \dfrac{\sum_{k \in G} (p_{ki}\tau_{kj} + p_{kj}\tau_{ik})}{T_i + T_j} & i \neq j, \\ 0 & i = j. \end{cases} \tag{29}$$

For uniform initialization, $\mu_i = 1/N$, we have

$$\tau_{ij} = \begin{cases} \dfrac{2 + \sum_{k \in G} \left(p_{ki}\tau_{kj} + p_{kj}\tau_{ik}\right)}{T_i + T_j} & i \neq j \\ 0 & i = j. \end{cases} \tag{30}$$

These recurrence relations hold for both directed and undirected weighted graphs.

3 Genetic algorithm

Here we describe the genetic algorithm developed by Möller et al. [7] to search for simple graphs with the largest ρ' and smallest ρ'/ρ° under temperature initialization, and smallest ρ' under uniform initialization.

We begin the process with m randomly generated connected simple graphs (unweighted, undirected, with no self-loops). We then assign k of those graphs as parents of the next generation by choosing graphs that optimize the desired property, such as the largest ρ'. Next, we recombine two of those parents into a new "child" graph. At this stage, we also mutate a small number of the child's edges through the inclusion of b mutations per individual per time step. This introduces enough randomness in the approach to make it less likely to get stuck in a local extremum.

The algorithm can be summarized as follows [7]:

1. Generate m connected Erdös-Rényi random graphs, with link probability p, to form the initial generation.

2. For each graph, calculate the property to be optimized (e.g. ρ' or ρ'/ρ°), and select the k most optimal graphs as parents for the next generation.

3. Generate m new "offspring" graphs, each created independently by the following procedure:

   (i) Sample two (not necessarily distinct) parents uniformly at random.

   (ii) For each (unordered) pair of vertices i and j, let the offspring inherit the edge weight $w_{ij} \in \{0, 1\}$ (i.e., absent or present) from one parent or the other, with equal probability.

   (iii) Randomly introduce mutations with fixed probability per edge, such that there are b mutations expected per offspring. Here, a mutation means that an edge is either eliminated or created.

   (iv) If a resulting graph is disconnected, repeat steps (i)–(iii), until a connected graph is obtained.

4. Repeat steps 2 and 3, n times.

The parameter values we used are given in Table 1.

Table 1: Parameters used in the genetic algorithm.

Parameter	Description	Value
m	number of random graphs per generation	400
p	Erdös-Rényi link probability	0.2
k	number of parents per generation	10
b	number of mutations per individual per time step	1
n	number of iterations	5000

4 Minimal absolute amplifier

In this and the following sections, we derive analytical results for particular graph families. We begin with the minimal absolute amplifier for temperature intialization, i.e. the "Bowtie" graph of Figure 12 of the main text. Computing ρ° and ρ' for this graph according to Eqs. (10), (22), and (29), we obtain the weak-selection expansion

$$\rho_G(1+\delta) = \frac{23}{144} + \frac{367}{864}\delta + \mathcal{O}(\delta^2). \tag{31}$$

To obtain the fixation probability for arbitrary mutant fitness r, we employed an algorithm of Cuesta et al. [8, 9], adapted for temperature initialization. This algorithm obtains a closed-form rational expression for $\rho_G(r)$ by evaluating this function at sufficiently many points. The necessary number of points is bounded by the number of distinct states of the evolutionary process (equivalently, the number of distinct 2-colorings of the graph), up to symmetry. This method yielded the expression $\rho_G(r) = \text{num}/\text{denom}$, where

$$\begin{aligned}\text{num} = {} & 49674643200r^{17} + 443672724240r^{16} + 1893000544452r^{15} \\ & + 5116448030706r^{14} + 9775218670398r^{13} + 13919892588853r^{12} \\ & + 15150505540668r^{11} + 12695464424106r^{10} + 8125168939102r^{9} \\ & + 3871299178035r^{8} + 1302413549640r^{7} + 277482979600r^{6} + 28278432000r^{5},\end{aligned} \tag{32a}$$

$$
\begin{aligned}
\text{denom} = {} & 49674643200r^{17} + 496107069840r^{16} + 2413909415772r^{15} \\
& + 7621969451652r^{14} + 17556513099030r^{13} + 31515614840412r^{12} \\
& + 46228915602810r^{11} + 57756165107886r^{10} + 63782585449398r^{9} \\
& + 63782585449398r^{8} + 57756165107886r^{7} + 46228915602810r^{6} \\
& + 31515614840412r^{5} + 17556513099030r^{4} + 7621969451652r^{3} \\
& + 2413909415772r^{2} + 496107069840r + 49674643200. \qquad (32b)
\end{aligned}
$$

Taylor expansion of $\rho_G(r)$ about $r = 1$ agrees with Eq. (31).

5 Star and complete bipartite graphs

We now turn to the complete bipartite graph K_{n_A,n_B}, which includes the Star as a special case. In this graph, the vertices are partitioned into two sets A and B, of respective sizes n_A and n_B. A given pair of vertices are joined by an edge if and only if they belong to different sets. The Star, S_n, is the special case $n_A = n,\ n_B = 1$ (or equivalently, $n_A = 1,\ n_B = n$).

For K_{n_A,n_B}, each vertex in set A has weighted degree $w_A = n_B$, while each vertex in set B has weighted degree $w_B = n_A$. The total inverse weighted degree is

$$
\tilde{W} = \frac{n_A}{n_B} + \frac{n_B}{n_A} = \frac{n_A^2 + n_B^2}{n_A n_B}. \qquad (33)
$$

The step probabilities are $p_{AB} = 1/n_B$ and $p_{BA} = 1/n_A$. Each A-vertex has temperature $T_A = n_B/n_A$, while each B-vertex has temperature $T_B = n_A/n_B$. We observe that in the case $n_A = n_B$, the graph is isothermal.

5.1 Temperature initialization

For temperature initialization, the neutral fixation probability, from Eq. (10), becomes

$$
\rho^\circ = \frac{1}{N\tilde{W}} \left(\frac{2n_A n_B}{w_A w_B} \right) = \frac{2n_A n_B}{(n_A + n_B)\left(n_A^2 + n_B^2\right)}. \qquad (34)
$$

Turning to the weak selection term ρ', there are three coalescence times to solve for: $\tau_{AA'}$ for distinct vertices in set A, $\tau_{BB'}$ for distinct vertices in set B, and τ_{AB} for vertices in different sets. The recurrence relation, Eq. (29),

becomes

$$\begin{aligned}
\tau_{AB} &= 1 + \frac{\left(\frac{n_B-1}{n_A}\right)\tau_{BB'} + \left(\frac{n_A-1}{n_B}\right)\tau_{AA'}}{\frac{n_B}{n_A} + \frac{n_A}{n_B}} \\
\tau_{AA'} &= 1 + \tau_{AB} \\
\tau_{BB'} &= 1 + \tau_{AB}.
\end{aligned}$$

Substituting the second and third equations into the first, and solving for τ_{AB}, yields

$$\tau_{AB} = \frac{2(n_A^2 + n_B^2) - (n_A + n_B)}{n_A + n_B}. \tag{35}$$

Substituting in Eq. (22) yields the first-order coefficient for fixation probability:

$$\rho' = \frac{1}{2N\tilde{W}}\left(\frac{2n_A n_B \tau_{AB}}{w_A w_B}\right) = \frac{n_A n_B \big(2(n_A^2 + n_B^2) - (n_A + n_B)\big)}{(n_A + n_B)^2\,(n_A^2 + n_B^2)}. \tag{36}$$

Setting $n_A = n$, $n_B = 1$ in Eqs. (34) and (36) yields the results for the Star S_n, for temperature initialization, reported in the main text.

We can compare to previous results [10, 11] for fixation probabilities on the bipartite graph with arbitrary mutant fitness, r. Following the solution of Monk et. al [11], we define the function

$$f_{a,b}(r) = r^{-(a+b)}\left(\frac{n_A + r n_B}{r n_A + n_B}\right)^{a-b}. \tag{37}$$

The fixation probability from a single mutant in set A or B, respectively, can then be written as [11]

$$\rho_A(r) = \frac{f_{1,0}(r) - 1}{f_{n_A,n_B}(r) - 1}, \qquad \rho_B(r) = \frac{f_{0,1}(r) - 1}{f_{n_A,n_B}(r) - 1}. \tag{38}$$

For the overall probability for temperature initialization, noting that $n_A T_A =$

n_B and $n_B T_B = n_A$, we obtain

$$\begin{aligned}
\rho_{K_{n_A,n_B}}(r) &= \frac{n_B\,\rho_A(r) + n_A\,\rho_B(r)}{n_A + n_B} \\
&= \frac{n_B f_{1,0}(r) + n_A f_{0,1}(r) - (n_A + n_B)}{(n_A + n_B)\left(f_{n_A,n_B}(r) - 1\right)} \\
&= \frac{\frac{n_B(n_A + r n_B)}{r(r n_A + n_B)} + \frac{n_A(r n_A + n_B)}{r(n_A + r n_B)} - (n_A + n_B)}{(n_A + n_B)\left(r^{-(n_A+n_B)}\left(\frac{n_A + r n_B}{r n_A + n_B}\right)^{n_A - n_B} - 1\right)}.
\end{aligned}$$

Taylor expansion gives

$$\begin{aligned}
\rho_{K_{n_A,n_B}}(r) = {} & \frac{2 n_A n_B}{(n_A + n_B)\left(n_A^2 + n_B^2\right)} \\
& + \frac{n_A n_B\big(2(n_A^2 + n_B^2) - (n_A + n_B)\big)}{(n_A + n_B)^2\left(n_A^2 + n_B^2\right)}\delta + \mathcal{O}(\delta^2), \quad (39)
\end{aligned}$$

which agrees with Eqs. (34) and (36).

Comparing to a well-mixed population of size $N = n_A + n_B$, we find

$$\rho' - \frac{N-1}{2N} = -\frac{(n_A - n_B)^2\big(n_A^2 + n_B^2 - (n_A + n_B)\big)}{2(n_A + n_B)^2(n_A^2 + n_B^2)} \quad (40)$$

$$\frac{\rho'}{\rho^\circ} - \frac{N-1}{2} = \frac{(n_A - n_B)^2}{2(n_A + n_B)}. \quad (41)$$

For $n_A \neq n_B$, we observe that $\rho' < (N-1)/(2N)$ while $\rho'/\rho^\circ > (N-1)/2$. We conclude that for temperature initialization, the complete bipartite graph K_{n_A,n_B}, with $n_A \neq n_B$, is an absolute suppressor and relative amplifier of weak selection.

5.2 Uniform initialization

For uniform initialization, the neutral fixation probability is $\rho^\circ = 1/N$, as on any graph. The recurrence relation for the τ_{ij}, Eq. (30), becomes

$$\begin{aligned}
\tau_{AB} &= \frac{2 + \left(\frac{n_B-1}{n_A}\right)\tau_{BB'} + \left(\frac{n_A-1}{n_B}\right)\tau_{AA'}}{\frac{n_B}{n_A} + \frac{n_A}{n_B}} \\
\tau_{AA'} &= \frac{n_A}{n_B} + \tau_{AB} \\
\tau_{BB'} &= \frac{n_B}{n_A} + \tau_{AB}.
\end{aligned}$$

Substituting the second two equations into the first and solving for τ_{AB} yields

$$\tau_{AB} = \frac{(n_A^2 + n_B^2)^2 - (n_A^3 + n_B^3)}{n_A + n_B}. \tag{42}$$

Substituting in Eq. (22) yields the first-order term for fixation probability:

$$\rho' = \frac{1}{2N\tilde{W}}\left(\frac{2n_A n_B \tau_{AB}}{w_A w_B}\right) = \frac{(n_A^2 + n_B^2)^2 - (n_A^3 + n_B^3)}{(n_A + n_B)^2\,(n_A^2 + n_B^2)}. \tag{43}$$

Setting $n_A = n,\ n_B = 1$ in Eq. (43) yields the result for the Star S_n, for uniform initialization, reported in the main text.

To compare to previous results [10, 11], we express the overall fixation probability for uniform initialization, via Eq. (38), as

$$\begin{aligned}
\rho_{K_{n_A,n_B}}(r) &= \frac{n_A\,\rho_A(r) + n_B\,\rho_B(r)}{n_A + n_B} \\
&= \frac{\frac{n_A(n_A+rn_B)}{r(rn_A+n_B)} + \frac{n_B(rn_A+n_B)}{r(n_A+rn_B)} - (n_A + n_B)}{(n_A + n_B)\left(r^{-(n_A+n_B)}\left(\frac{n_A+rn_B}{rn_A+n_B}\right)^{n_A-n_B} - 1\right)}.
\end{aligned}$$

Taylor expansion gives

$$\rho_{K_{n_A,n_B}}(1+\delta) = \frac{1}{n_A + n_B} + \frac{(n_A^2 + n_B^2)^2 - (n_A^3 + n_B^3)}{(n_A + n_B)^2\,(n_A^2 + n_B^2)}\delta + \mathcal{O}(\delta^2), \tag{44}$$

which agrees with Eq.(43).

Comparing to the well-mixed population of size $N = n_A + n_B$, we find

$$\rho' - \frac{N-1}{2N} = \frac{(n_A - n_B)^2\big(n_A^2 + n_B^2 - (n_A + n_B)\big)}{2(n_A + n_B)^2(n_A^2 + n_B)^2}. \tag{45}$$

For $n_A \neq n_B$, we observe that $\rho' > (N-1)/(2N)$, meaning that for uniform initialization, K_{n_A,n_B} is an amplifier of weak selection as long as $n_A \neq n_B$.

Curiously, comparing Eqs. (41) and (45), we find that the difference $\rho' - (N-1)/(2N)$ for uniform and temperature initialization are opposites of each other. While this result is intriguing, its significance is unclear.

6 Fan

The Fan graph (Fig 12 of the main text) consists of a hub vertex attached to n blades. Each blade is comprised of m vertices. Within a given blade, each vertex is connected to every other vertex with weight 1; in symbols, $w_{BB'} = 1$. The hub is connected to every vertex in the blades with an edge weight of ϵ; in symbols, $w_{HB} = \epsilon$. There are no connections between different blades. The total population size is $N = mn + 1$.

6.1 Temperature initialization

6.1.1 Weighted Degrees and Step Probabilities

The weighted degrees are $w_H = mn\epsilon$ for the hub and $w_B = m - 1 + \epsilon$ for each blade vertex. The step probabilities are

- $p_{HB} = \frac{1}{mn}$ from the hub to any given blade vertex,
- $p_{BH} = \frac{\epsilon}{m-1+\epsilon}$ from any given blade vertex to the hub,
- $p_{BB'} = \frac{1}{m-1+\epsilon}$ from one vertex to any other given vertex in the same blade.

The total inverse weighted degree is

$$\tilde{W} = w_H^{-1} + mnw_B^{-1} = \frac{1}{mn\epsilon} + \frac{mn}{(m-1)+\epsilon}. \tag{46}$$

The temperatures are

$$T_H = mnp_{BH} = \frac{mn\epsilon}{m-1+\epsilon}. \tag{47}$$

for the hub, and

$$T_B = p_{HB} + (m-1)p_{BB`} = \frac{1}{mn} + \frac{m-1}{m-1+\epsilon}. \tag{48}$$

for each blade vertex.

6.1.2 Fixation probability

For temperature initialization, applying Eq. (10) gives a neutral fixation probability of

$$\begin{aligned}
\rho^\circ &= \frac{1}{N\tilde{W}}\left(2mn\frac{w_{BH}}{w_B w_H} + mn(m-1)\frac{w_{BB`}}{w_B^2}\right) \\
&= \frac{1}{(mn+1)\left(\frac{1}{mn\epsilon} + \frac{mn}{(m-1+\epsilon)}\right)}\left(\frac{2mn\epsilon}{(mn\epsilon)(m-1+\epsilon)} + \frac{mn(m-1)}{(m-1+\epsilon)^2}\right) \\
&= \frac{mn\epsilon(m-1+2\epsilon)(mn+2)}{(m-1+\epsilon)(mn+1)(m-1+\epsilon(m^2n^2+1))}.
\end{aligned} \tag{49}$$

To find the first-order term, ρ', we must compute three coalescence times: τ_{HB} for the hub and a blade vertex, $\tau_{BB'}$ for two distinct vertices on the same blade, and $\tau_{BB''}$ for two vertices on different blades. These are determined by the following system of equations:

$$\tau_{HB} = 1 + \frac{(m-1)p_{BH}\tau_{BB'} + m(n-1)p_{BH}\tau_{BB''} + (m-1)p_{BB'}\tau_{HB}}{T_H + T_B} \tag{50a}$$

$$\tau_{BB'} = 1 + \frac{p_{HB}\tau_{HB} + (m-2)p_{BB'}\tau_{BB'}}{T_B} \tag{50b}$$

$$\tau_{BB''} = 1 + \frac{p_{HB}\tau_{HB} + (m-1)p_{BB'}\tau_{BB''}}{T_B}. \tag{50c}$$

Solving this system and substituting in Eq. (22), we obtain the first-order term, ρ'

$$\rho' = \frac{1}{2N\tilde{W}}\left(2mn\frac{w_{BH}}{w_B w_H}\tau_{HB} + mn(m-1)\frac{w_{BB`}}{w_B^2}\tau_{BB'}\right) = \frac{\text{num}}{\text{denom}}, \tag{51}$$

where

$$\begin{aligned}\text{num} = mn\epsilon\big(2(m-1+\epsilon)^3 + 2mn(m-1+\epsilon)^2(2m-1-\epsilon) \\ + 2m^2n^2(m-1+\epsilon)(2\epsilon^2-\epsilon+m^2-m) \\ + 2m^4n^3\epsilon(m+2\epsilon-1+nm^2-n))\big).\end{aligned} \tag{52}$$

$$\begin{aligned}\text{denom} = 2(m-1+\epsilon)(mn+1)(m-1+\epsilon(m^2n^2+1))((m-1+\epsilon)^2 \\ + mn(1+\epsilon)(m-1+\epsilon) + m^3n^2\epsilon).\end{aligned} \tag{53}$$

6.1.3 Behavior in terms of ϵ

We now analyze how ρ and ρ' vary with ϵ. As $\epsilon \to 0$, we have the following limits:

$$\lim_{\epsilon\to 0} \rho^\circ = \lim_{\epsilon\to 0} \rho' = 0, \tag{54}$$

$$\lim_{\epsilon\to 0} \frac{\rho'}{\rho^\circ} = \frac{(mn+1)\,(m^2n+m-1)}{(mn+2)(mn+m-1)}. \tag{55}$$

As $\epsilon \to \infty$, we have

$$\lim_{\epsilon\to\infty} \rho^\circ = \frac{2mn}{(1+mn)(1+(mn)^2)}, \tag{56}$$

$$\lim_{\epsilon\to\infty} \rho' = \frac{mn(1-mn+2(mn)^2)}{(1+mn)^2(1+(mn)^2)}. \tag{57}$$

These limiting ρ° and ρ' values are the same as for the star with mn leaves, S_{mn}. This makes sense, because in the $\epsilon \to \infty$ limit, the blade-to-blade weights are negligible in comparison to the hub-to-blade weights, making the Fan equivalent to a Star.

We now show that the Fan displays all three classifications of behavior, depending on the value of ϵ.

Theorem 1. *The Fan $F_{n,m}$ is*

(a) An absolute and relative suppressor for $0 < \epsilon < (m-1)/(mn-1)$,

(b) An absolute and relative amplifier for $(m-1)/(mn-1) < \epsilon < \epsilon^*$, *where ϵ^* is a particular cubic root (depending on m and n), and*

(c) *A relative amplifier and absolute suppressor for* $\epsilon > \epsilon^*$.

Proof. We first prove the statements about absolute amplification and suppression, by examining the bevavior of ρ'. From Eqs. (55) and (57), we have that $\rho' < (N-1)/(2N)$ for all sufficiently small or sufficiently large ϵ. To determine the behavior between these limits, we set $\rho' = (N-1)/(2N) = (mn)/(2mn+2)$. Upon substituting and factoring with the aid of Mathematica, we find that this equation is equivalent to

$$\big((mn-1) - (m-1)\epsilon\big)p(\epsilon) = 0, \tag{58}$$

where $p(\epsilon)$ is the cubic

$$\begin{aligned} p(\epsilon) &= \epsilon^3(mn-1)^2 + \epsilon^2\left(mn(mn-1)\left(m^2n-1\right) + m - 1\right) \\ &- \epsilon(m-1)\left(m^3n^3 - 2m^2n + 2mn + m - 1\right) - (m-1)^2(mn+m-1). \end{aligned} \tag{59}$$

By Descartes' Rule of Signs, $p(\epsilon)$ has exactly one positive root (counting multiplicity); call it ϵ^*. Therefore, the left-hand side of Eq. (58) has single roots at $\epsilon = (m-1)/(mn-1)$ and $\epsilon = \epsilon^*$. We also observe that $p(0) < 0$, and

$$p\left(\frac{m-1}{mn-1}\right) = -\frac{m^4(m-1)^2n^3(n-1)}{(mn-1)^2} < 0. \tag{60}$$

Since $p(\epsilon)$ has no positive roots aside from ϵ^*, we must have $\epsilon^* > (m-1)/(mn-1)$. It follows that $\rho' > (N-1)/(2N)$ only for $(m-1)/(mn-1) < \epsilon < \epsilon^*$. We conclude that the Fan is an absolute suppressor for $0 < \epsilon < (m-1)/(mn-1)$, an absolute amplifier for $(m-1)/(mn-1) < \epsilon < \epsilon^*$, and an absolute suppressor again for $\epsilon > \epsilon^*$.

We now turn to the claims regarding relative amplification and suppression, by examining the behavior of ρ'/ρ°. Comparing the $\epsilon \to 0$ limit to the well-mixed value of $(N-1)/2 = nm/2$, we find (after some algebra)

$$\lim_{\epsilon\to 0}\frac{\rho'}{\rho^\circ} - \frac{mn}{2} = -\frac{m^2n(n-2)(mn+1) + m\left(m^2n^2-2\right) + 2}{2(mn+2)(mn+m-1)}. \tag{61}$$

Recalling that $m, n \geq 2$, we see that the right-hand side above is negative; thus $\rho'/\rho^\circ < (N-1)/2$ for all sufficiently small ϵ. Since the $\epsilon \to \infty$ limit recovers the results for the Star, we have that $\rho'/\rho^\circ > (N-1)/2$ for all sufficiently large ϵ. To determine the behavior between these limits, we set

$\rho'/\rho^\circ = mn/2$. Upon substituting and factoring with the aid of Mathematica, this equation becomes equivalent to

$$\big((mn-1)\epsilon - (m-1)\big)q(\epsilon) = 0, \tag{62}$$

where $q(\epsilon)$ is the quadratic

$$q(\epsilon) = 2\epsilon^2\,(mn-1) + \epsilon\big(m(mn-2)(mn+n+2)+4\big) \\ +(m-1)\big(m^2(n-2)(mn^2+mn+2m+n)+2(m-1)(2m^2+2m+1)+4\big). \tag{63}$$

All coefficients of $q(\epsilon)$ are positive, so it has no positive roots. This shows that $\rho'/\rho^\circ - mn/2$ changes sign at most once. Combining with our information about the $\epsilon \to 0$ and $\epsilon \to \infty$ limits shows that the $F_{n,m}$ is a relative suppressor for $0 < \epsilon < (m-1)/(mn-1)$ and a relative amplifier for $\epsilon > (m-1)/(mn-1)$. This completes the proof. □

6.1.4 Asymptotic behavior as $n \to \infty$

Here we examine the behavior of ρ' and $N\rho^\circ$ in the $n \to \infty$ limit. We note that this limit is taken after the weak-selection limit; thus this is a wN-limit in the sense of Ref. [12]. In this limit, a graph is an absolute amplifier (respectively, absolute suppressor) if the limiting value of ρ' is greater than (respectively, less than) 1/2; a graph is a relative amplifier (respectively, relative suppressor) if the limiting value of $\rho'/(N\rho^\circ)$ is greater than (respectively, less than) 1/2.

We consider three possible ways that ϵ could scale with n. First, if ϵ is constant with respect to n, we obtain

$$\lim_{n\to\infty} \rho' = \frac{m^2-1}{2m(m-1+\epsilon)} \tag{64a}$$

$$\lim_{n\to\infty} N\rho^\circ = \frac{m-1}{m-1+\epsilon} \tag{64b}$$

$$\lim_{n\to\infty} \frac{\rho'}{N\rho^\circ} = \frac{m+1}{2m}. \tag{64c}$$

We observe that $\lim_{n\to\infty} \rho' > 1/2$ when $\epsilon < (m-1)/m$, and $\lim_{n\to\infty} \rho' < 1/2$ when $\epsilon > (m-1)/m$. On the other hand, $\lim_{n\to\infty} \rho'/(N\rho^\circ)$ is independent of ϵ and is always greater than 1/2.

Second, we suppose that ϵ scales inversely with n, by setting $\epsilon = kn^{-1}$. This gives

$$\lim_{n\to\infty} \rho' = \frac{km(m+1)}{2\left(km^2+m-1\right)} \tag{65a}$$

$$\lim_{n\to\infty} N\rho^\circ = 1 \tag{65b}$$

$$\lim_{n\to\infty} \frac{\rho'}{N\rho^\circ} = \frac{km(m+1)}{2\left(km^2+m-1\right)}. \tag{65c}$$

With this scaling, $\lim_{n\to\infty} \rho'$ and $\lim_{n\to\infty} \rho'/(N\rho^\circ)$ are both greater than 1/2 when $k > (m-1)/m$, or equivalently $\epsilon > (m-1)/(mn)$; the opposite is true when $\epsilon < (m-1)/(mn)$.

Finally, we consider the inverse square scaling $\epsilon = kn^{-2}$, which gives

$$\lim_{n\to\infty} \rho' = 0 \tag{66a}$$

$$\lim_{n\to\infty} N\rho^\circ = \frac{km^2}{km^2+m-1} \tag{66b}$$

$$\lim_{n\to\infty} \frac{\rho'}{N\rho^\circ} = 0. \tag{66c}$$

Putting these results together, we conclude that, asymptotically as $n \to \infty$, the Fan is an absolute and relative suppressor for $0 < \epsilon < (m-1)/(mn)$, an absolute and relative amplifier for $(m-1)/(mn) < \epsilon < (m-1)/m$, and a relative amplifier and absolute suppressor for $\epsilon > (m-1)/m$.

6.1.5 Maximizing ρ'

Eqs. (64a) and (65a) suggest that ρ' is maximized for a scaling of ϵ that is between n^0 and n^{-1}, as $n \to \infty$. To determine the precise scaling that maximizes ρ', we take the derivative of ρ' with respect to ϵ. The numerator of $d\rho'/d\epsilon$ can be written as

$$(m-1)m^8n^8\epsilon^2\left(-m^2(m+1)n\epsilon^2 - 2(m+1)\epsilon^3 - (m-1)\epsilon^2 + (m-1)^2(m+1)\right) + \mathcal{O}(n^7) \qquad (n\to\infty). \tag{67}$$

Since the maximizing ϵ should scale between n^0 and n^{-1}, we suppose that $\lim_{n\to\infty}\epsilon = 0$ and $\lim_{n\to\infty}(n\epsilon) = \infty$. Applying these limits to Eq. (67), we find that, asymptotically as $n \to \infty$, $d\rho'/d\epsilon = 0$ reduces to

$$-m^2(m+1)n\epsilon^2 + (m-1)^2(m+1) = 0. \tag{68}$$

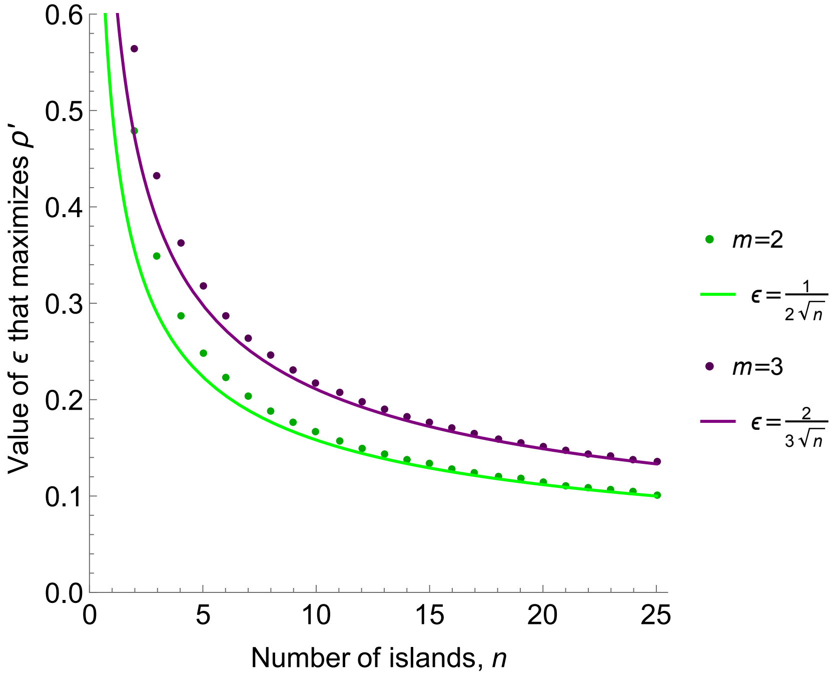

Figure S1: **Maximizing ρ' for the Fan.** The values of ϵ that maximize ρ' for the Fan graphs $F_{n,2}$ and $F_{n,3}$, calculated numerically in Mathematica, are plotted (dots) against the number of blades n. Curves show the approximated maximand $\epsilon = (m-1)/(m\sqrt{n})$, which is asymptotically exact in the $n \to \infty$ limit.

Solving for ϵ, we obtain

$$\epsilon = \frac{m-1}{m\sqrt{n}}. \tag{69}$$

Thus the ϵ that maximizes ρ' is asymptotically $\epsilon = (m-1)/(m\sqrt{n})$ as $n \to \infty$. With this scaling of ϵ with respect to n, we obtain

$$\lim_{n\to\infty} \rho' = \frac{m+1}{2m} \tag{70}$$

$$\lim_{n\to\infty} N\rho^\circ = 1. \tag{71}$$

Figure S1 shows that $(m-1)/(m\sqrt{n})$ accurately estimates the maximizing values of ϵ for finite n.

6.2 Uniform initialization

6.2.1 Derivation of ρ'

We now consider the Fan with uniform initialization. Eq. (30) gives us the system of equations

$$\tau_{HB} = \frac{2 + (m-1)p_{BH}\tau_{BB'} + m(n-1)p_{BH}\tau_{BB''} + (m-1)p_{BB'}\tau_{HB}}{T_H + T_B} \tag{72a}$$

$$\tau_{BB'} = \frac{1 + p_{HB}\tau_{HB} + (m-2)p_{BB'}\tau_{BB'}}{T_B} \tag{72b}$$

$$\tau_{BB''} = \frac{1 + p_{HB}\tau_{HB} + (m-1)p_{BB'}\tau_{BB''}}{T_B}. \tag{72c}$$

Solving and substituting in Eq. (22), we obtain

$$\rho'_{\text{unif}} = \frac{\text{num}}{\text{denom}}, \tag{73a}$$

with

$$\begin{aligned} \text{num} = m^2n^2\epsilon \big(&m^3(m+1)n^3\epsilon + 2m^2n^2(\epsilon-1)\epsilon \\ &+mn(3m-2\epsilon+1)(m+\epsilon-1) + 4(m+\epsilon-1)^2\big) \end{aligned} \tag{73b}$$

$$\begin{aligned} \text{denom} = 2(mn+1)&\left(m^2n^2\epsilon + m + \epsilon - 1\right) \\ &\times \left(m^3n^2\epsilon + mn(\epsilon+1)(m+\epsilon-1) + (m+\epsilon-1)^2\right). \end{aligned} \tag{73c}$$

6.2.2 Behavior in terms of ϵ

We observe that $\lim_{\epsilon\to 0} \rho' = 0$. This indicates that the Fan with uniform initialization is a suppressor of weak selection for all sufficiently small ϵ. As $\epsilon \to \infty$, the ρ' value approaches that of the Star S_{mn}, which is an amplifier of weak selection. Therefore the Fan in an amplifier of weak selection for all sufficiently large ϵ.

To determine the behavior between these limits, we set $\rho' = (N-1)/(2N) = (mn)/(2(mn+1))$. Upon substituting and factoring with the aid of Mathematica, this equation becomes equivalent to

$$\big((mn-1)\epsilon - (m-1)\big)q(\epsilon) = 0, \tag{74}$$

where $q(\epsilon)$ is the quadratic

$$q(\epsilon) = \epsilon^2(mn-1)^2 \\ +\epsilon\left(mn\left((m-1)^2(n-1)n + (m-1)(n-2)(2n+1) + (n-1)^2\right) + 2(m-1)\right) \\ + (m-1)(mn+m-1). \quad (75)$$

All coefficients in $q(\epsilon)$ are positive, so there are no positive roots. Therefore, $\rho' = (N-1)/(2N)$ only in the case $\epsilon = (m-1)/(mn-1)$, at which value the Fan is isothermal. We conclude that the Fan with uniform initialization is a suppressor of weak selection for $0 < \epsilon < (m-1)/(mn-1)$, and an amplifier of weak selection for $\epsilon > (m-1)/(mn-1)$.

7 Cartwheel

The Cartwheel graph $CW_{n,m,h}$ (Fig 9 of the main text) consists of a hub with h vertices, and $n \leq h$ islands with m vertices each. One vertex in each island, and n vertices in the hub, are designated as "connectors". We use the following vertex labels: H for non-connector hub, $\tilde{H}$ for connector hub, I for non-connector island, and $\tilde{I}$ for connector island.

Within the hub, all edge weights are 1; that is, $w_{HH} = w_{H\tilde{H}} = w_{\tilde{H}\tilde{H}} = 1$. Within an island, all edge weights are 1 as well: $w_{II} = w_{I\tilde{I}} = 1$. Each hub connector is connected to a single island connector by an edge of weight ϵ: $w_{\tilde{H}\tilde{I}} = \epsilon$.

The step probabilities are as follows:

- One non-connector hub to another: $p_{HH'} = \frac{1}{h-1}$.
- Non-connector hub to connector hub: $p_{H\tilde{H}} = \frac{1}{h-1}$.
- Connector hub to non-connector hub: $p_{\tilde{H}H} = \frac{1}{h-1+\epsilon}$.
- One connector hub to another $p_{\tilde{H}\tilde{H}'} = \frac{1}{h-1+\epsilon}$.
- Distinct non-connector island vertices in the same island: $p_{II'} = \frac{1}{m-1}$.
- Non-connector island vertex to connector vertex in the same island: $p_{I\tilde{I}} = \frac{1}{m-1}$.
- Connector island vertex to non-connector vertex in the same island: $p_{\tilde{I}I} = \frac{1}{m-1+\epsilon}$.

- Connector hub vertex to corresponding connector island vertex: $p_{\tilde{H}\tilde{I}} = \frac{\epsilon}{h-1+\epsilon}$.
- Connector island vertex to corresponding connector hub vertex: $p_{\tilde{I}\tilde{H}} = \frac{\epsilon}{m-1+\epsilon}$.

The temperatures of the vertices are as follows:

$$T_H = (n-h-1)p_{HH'} + np_{\tilde{H}H} = \frac{h-n-1}{h-1} + \frac{n}{h-1+\epsilon} \tag{76a}$$

$$\begin{aligned} T_{\tilde{H}} &= (n-1)p_{\tilde{H}\tilde{H}'} + (h-n)p_{H\tilde{H}} + p_{\tilde{I}\tilde{H}} \\ &= \frac{n-1}{h-1+\epsilon} + \frac{h-n}{h-1} + \frac{\epsilon}{m-1+\epsilon} \end{aligned} \tag{76b}$$

$$T_I = (m-2)p_{II'} + p_{\tilde{I}I} = \frac{m-2}{m-1} + \frac{1}{m-1+\epsilon} \tag{76c}$$

$$T_{\tilde{I}} = (m-1)p_{I\tilde{I}} + p_{\tilde{H}\tilde{I}} = 1 + \frac{\epsilon}{h-1+\epsilon}. \tag{76d}$$

7.1 Neutral fixation probability

To obtain the neutral fixation probability, ρ°, we first compute the sum of inverse weighted degrees:

$$\tilde{W} = \frac{(m-1)n}{w_I} + \frac{n}{w_{\tilde{I}}} + \frac{h-n}{w_H} + \frac{n}{w_{\tilde{H}}} \tag{77}$$

$$= n + \frac{n}{m-1+\epsilon} + \frac{h-n}{h-1} + \frac{n}{m-1+\epsilon}. \tag{78}$$

We then find ρ° from Eq. (10):

$$\begin{aligned} \rho^\circ = \frac{1}{N\tilde{W}} \Bigg(&\frac{2n\epsilon}{w_{\tilde{I}}w_{\tilde{H}}} + \frac{2n(m-1)}{w_{\tilde{I}}w_I} + \frac{2n(h-n)}{w_{\tilde{H}}w_H} \\ &+ \frac{(h-n)(h-n-1)}{w_H^2} + \frac{(m-1)(m-2)n}{w_I^2} + \frac{n(n-1)}{w_{\tilde{H}}^2} \Bigg) \end{aligned} \tag{79}$$

$$= \frac{\frac{2\epsilon n}{(\epsilon+h-1)(\epsilon+m-1)} + \frac{2n(h-n)}{(h-1)(\epsilon+h-1)} + \frac{(n-1)n}{(\epsilon+h-1)^2} + \frac{2n}{\epsilon+m-1} + \frac{(h-n-1)(h-n)}{(h-1)^2} + \frac{(m-2)n}{m-1}}{(h+mn)\left(\frac{n}{\epsilon+h-1} + \frac{n}{\epsilon+m-1} + \frac{h-n}{h-1} + n\right)}. \tag{80}$$

7.2 Weak selection

We now obtain the first-order term, ρ', giving the effect of weak selection. For this, we must solve for the pairwise coalescence times τ_{ij} according to Eq. (29). There are fourteen distinct coalescence times to consider. We explicate the recurrence equations for the first two of these; the rest are constructed similarly.

- Two distinct non-connector hub vertices:
$$\tau_{HH'} = 1 + \frac{(h-n-2)p_{HH}\tau_{HH'} + np_{\tilde{H}H}\tau_{H\tilde{H}}}{T_H}. \tag{81a}$$
The first term in the numerator corresponds to the $h-n-2$ other hub non-connector vertices that could replace the one of the two in question, with step probability $p_{HH'}$ for each. The second term in the numerator corresponds to the n connector hub vertices that could replace either of the non-connector hub vertices in question, with step probability $p_{\tilde{H}H}$ for each. Since the two vertices in question are equivalent to each other, a factor of two cancels out in the numerator and denominator.

- Non-connector hub vertex and connector hub vertex:
$$\tau_{H\tilde{H}} = 1 + \frac{1}{T_H + T_{\tilde{H}}}\big((h-n-1)p_{HH'}\tau_{H\tilde{H}} + (n-1)p_{\tilde{H}H}\tau_{\tilde{H}\tilde{H}'} \\ + (n-1)p_{\tilde{H}\tilde{H}}\tau_{H\tilde{H}} + (h-n-1)p_{H\tilde{H}}\tau_{HH'} + p_{\tilde{I}\tilde{H}}\tau_{\tilde{I}H}\big). \tag{81b}$$
The first two terms in parentheses correspond to the non-connector hub vertex being replaced by, respectively, a distinct non-connector hub vertex and a distinct connector hub vertex. The next three correspond to the connector hub vertex being replaced by, respectively, a distinct connector hub vertex, a distinct non-connector hub vertex, and the corresponding connector island vertex.

- Two distinct connector hub vertices:
$$\tau_{\tilde{H}\tilde{H}'} = 1 + \frac{(n-2)p_{\tilde{H}\tilde{H}'}\tau_{\tilde{H}\tilde{H}'} + p_{\tilde{I}\tilde{H}}\tau_{\tilde{I}\tilde{H}'} + (h-n)p_{H\tilde{H}}\tau_{H\tilde{H}}}{T_{\tilde{H}}}. \tag{81c}$$

- Connector island vertex and non-connector hub vertex:
$$\tau_{\tilde{I}H} = 1 + \frac{1}{T_{\tilde{I}} + T_H}\big(p_{\tilde{H}\tilde{I}}\tau_{H\tilde{H}} + (m-1)p_{I\tilde{I}}\tau_{IH} \\ + (n-1)p_{\tilde{H}H}\tau_{\tilde{I}\tilde{H}'} + p_{\tilde{H}H}\tau_{\tilde{I}\tilde{H}} + (h-n-1)p_{HH'}\tau_{\tilde{I}H}\big). \tag{81d}$$

- Connector island vertex and corresponding connector hub vertex:

$$\tau_{\tilde{I}\tilde{H}} = 1 + \frac{(m-1)p_{I\tilde{I}}\tau_{I\tilde{H}} + (n-1)p_{\tilde{H}\tilde{H}'}\tau_{\tilde{I}\tilde{H}'} + (h-n)p_{H\tilde{H}}\tau_{\tilde{I}H}}{T_{\tilde{I}} + T_{\tilde{H}}}. \tag{81e}$$

- Connector island vertex and corresponding connector hub vertex:

$$\tau_{\tilde{I}\tilde{H}'} = 1 + \frac{1}{T_{\tilde{I}} + T_{\tilde{H}}}\big((m-1)p_{I\tilde{I}}\tau_{I\tilde{H}'} + p_{\tilde{H}\tilde{I}}\tau_{\tilde{H}\tilde{H}'} + p_{\tilde{I}\tilde{H}}\tau_{\tilde{I}\tilde{I}'} + (n-2)p_{\tilde{H}\tilde{H}'}\tau_{\tilde{I}\tilde{H}'} + p_{\tilde{H}\tilde{H}'}\tau_{\tilde{I}\tilde{H}} + (h-n)p_{H\tilde{H}}\tau_{\tilde{I}H}\big). \tag{81f}$$

- Two distinct non-connector island vertices on the same island:

$$\tau_{II'} = 1 + \frac{p_{\tilde{I}I}\tau_{I\tilde{I}} + (m-3)p_{II'}\tau_{II'}}{T_I}. \tag{81g}$$

- Connector and non-connector vertices on the same island:

$$\tau_{I\tilde{I}} = 1 + \frac{p_{\tilde{H}\tilde{I}}\tau_{I\tilde{H}} + (m-2)p_{I\tilde{I}}\tau_{II'} + (m-2)p_{II'}\tau_{I\tilde{I}}}{T_I + T_{\tilde{I}}}. \tag{81h}$$

- Two non-connector island vertices on different islands:

$$\tau_{II''} = 1 + \frac{p_{\tilde{I}I}\tau_{I\tilde{I}'} + (m-2)p_{II'}\tau_{II''}}{T_I}. \tag{81i}$$

- Non-connector vertex on one island and connector vertex on a different island:

$$\tau_{I\tilde{I}'} = 1 + \frac{(m-2)p_{II'}\tau_{I\tilde{I}'} + p_{\tilde{I}I}\tau_{\tilde{I}\tilde{I}'} + p_{\tilde{H}\tilde{I}}\tau_{I\tilde{H}'} + (m-1)p_{I\tilde{I}}\tau_{II''}}{T_I + T_{\tilde{I}}} \tag{81j}$$

- Two connector island vertices on different islands:

$$\tau_{\tilde{I}\tilde{I}'} = 1 + \frac{p_{\tilde{H}\tilde{I}}\tau_{\tilde{I}\tilde{H}'} + (m-1)p_{I\tilde{I}}\tau_{I\tilde{I}'}}{T_{\tilde{I}}}. \tag{81k}$$

- Non-connector island and hub vertices

$$\tau_{IH} = 1 + \frac{1}{T_I + T_H}\big(p_{\tilde{I}I}\tau_{\tilde{I}H} + (m-2)p_{II'}\tau_{IH} + (n-1)p_{\tilde{H}H}\tau_{I\tilde{H}'} + p_{\tilde{H}H}\tau_{I\tilde{H}} + (h-n-1)p_{HH'}\tau_{IH}\big). \tag{81l}$$

- Non-connector island vertex and corresponding hub connector vertex:

$$\tau_{I\tilde{H}} = 1 + \frac{1}{T_I + T_{\tilde{H}}} \big(p_{\tilde{I}I} \tau_{\tilde{I}\tilde{H}} + (m-2) p_{II'} \tau_{I\tilde{H}} + p_{\tilde{I}\tilde{H}} \tau_{I\tilde{I}} \\ + (n-1) p_{\tilde{H}\tilde{H}'} \tau_{I\tilde{H}'} + (h-n) p_{H\tilde{H}} \tau_{IH} \big). \quad (81m)$$

- Non-connector island vertex and non-corresponding hub connector vertex:

$$\tau_{I\tilde{H}'} = 1 + \frac{1}{T_I + T_{\tilde{H}}} \big(p_{\tilde{I}I} \tau_{\tilde{I}\tilde{H}'} + (m-2) p_{II'} \tau_{I\tilde{H}'} + p_{\tilde{I}\tilde{H}} \tau_{I\tilde{I}'} \\ + (n-2) p_{\tilde{H}\tilde{H}'} \tau_{I\tilde{H}'} + p_{\tilde{H}\tilde{H}'} \tau_{I\tilde{H}} + (h-n) p_{H\tilde{H}} \tau_{IH} \big). \quad (81n)$$

Some amendments to these equations are required in special cases. For example, in the case $m = 2$, there are no pairs II', thus the equation for $\tau_{II'}$ is eliminated, as are all terms involving $\tau_{II'}$. Likewise, if $n = h$, then there are no non-connector vertices (H), and so the equations for $\tau_{HH'}$, $\tau_{H\tilde{H}}$, τ_{IH}, and $\tau_{\tilde{I}H}$ are eliminated, as are any terms involving these quantities.

We have solved the above system of equations analytically using Mathematica. The final weak-selection coefficient ρ' for the Cartwheel $CW_{n,m,h}$ is then obtained from Eq. (22) as

$$\rho' = \frac{1}{2N\tilde{W}} \left(\frac{2n\epsilon}{w_{\tilde{I}} w_{\tilde{H}}} \tau_{\tilde{I}\tilde{H}} + \frac{2n(m-1)}{w_{\tilde{I}} w_I} \tau_{I\tilde{I}} + \frac{2n(h-n)}{w_{\tilde{H}} w_H} \tau_{\tilde{H}H} \right. \\ \left. + \frac{(h-n)(h-n-1)}{w_H^2} \tau_{HH'} + \frac{(m-1)(m-2)n}{w_I^2} \tau_{II'} + \frac{n(n-1)}{w_{\tilde{H}}^2} \tau_{\tilde{H}\tilde{H}'} \right). \quad (82)$$

The full expression for ρ' in terms of n, m, h, and ϵ is too lengthy to include here. However, it can be evaluated in Mathematica and we have used it to create plots in the main text.

7.3 Full selection with $\epsilon \to 0$

Here we derive the fixation probability on the Cartwheel graph for arbitrary mutant fitness r, in the limit as the hub-to-island weight ϵ goes to zero. This limit induces a separation of timescales. Fixation of one type or the other within the hub or within an island occurs on the fast timescale. Changes in which subpopulations (hub or islands) are fixed for residents or mutants occur on the slow timescale.

7.3.1 Derivation of fixation probability

To calculate fixation probability in the $\epsilon \to 0$ limit, we employ an approach developed by Allen et al. [13] for a different graph structure and update rule, building on a method that Hadjichrisanthou et al. [14] employed for the Star graph.

This method relies on a separation of timescales [15], in the Markov chain representing the death-Birth process on the Cartwheel, as $\epsilon \to 0$. To achieve this timescale separation, we rescale time by a factor of $1/\epsilon$, and then take the limit as $\epsilon \to 0$. The result is a continuous-time Markov chain in which some transitions occur instantaneously. Specifically, let $p_{\mathbf{x}\to\mathbf{y}}$ denote the transition probability from state $\mathbf{x}$ to state $\mathbf{y}$ in the original Markov chain. If $\lim_{\epsilon\to 0^+} p_{\mathbf{x}\to\mathbf{y}} > 0$, then the transition $\mathbf{x} \to \mathbf{y}$ occurs instantaneously in the time-rescaled Markov chain, with probability equal to $\lim_{\epsilon\to 0^+} p_{\mathbf{x}\to\mathbf{y}}$. Otherwise, the transition $\mathbf{x} \to \mathbf{y}$ occurs at rate $q_{\mathbf{x}\to\mathbf{y}} = \lim_{\epsilon\to 0^+} \frac{p_{\mathbf{x}\to\mathbf{y}}}{\epsilon}$. Informally, we say that instantaneous transitions occur "on the fast timescale", while transitions with finite rates occur "on the slow timescale".

With this rescaling, fixation within the hub or within each island occurs instantaneously (i.e. on the fast timescale). Thus the only states with nonzero holding times are those for which each subpopulation (hub or island) contains only one type. These states can be denoted as (M, k) or (R, k), where the first entry is the type of the hub, and the second is the number k of islands that are fixed for mutants, $0 \le k \le n$. We can therefore reduce the state space of the time-rescaled Markov chain to states of the form (M, k) or (R, k), $0 \le k \le n$.

We now identify the rates of transition between these states, starting with the rate $Q_{(M,k)\to(M,k+1)}$ from (M, k) to $(M, k+1)$. This transition involves the mutant type taking over an island previously controlled by residents, given that the hub is already fixed for mutants. Two steps are required for this transition. First, a mutant at a connector hub vertex must reproduce onto an island that is fixed for residents. Noting that there are $n - k$ resident islands—and thus $n - k$ mutants that are positioned to reproduce onto these islands—the probability of this event occurring in the original Markov chain is

$$\left(\frac{(n-k)r}{(h+km)r+(n-k)m}\right)\left(\frac{\epsilon}{\epsilon+h-1}\right).$$

Therefore, in the time-rescaled Markov chain, this event occurs at rate

$$\lim_{\epsilon \to 0^+} \left[\left(\frac{1}{\epsilon}\right) \left(\frac{(n-k)r}{(h+km)r+(n-k)m}\right) \left(\frac{\epsilon}{\epsilon+h-1}\right) \right] = \left(\frac{n-k}{(h+km)r+(n-k)m}\right) \left(\frac{1}{h-1}\right).$$

Second, to complete the transition to state $(M, k+1)$, the mutant type must reach fixation on the island in question. This occurs instantaneously (on the fast timescale), and has probability $(1-r^{-1})/(1-r^{-m})$. Putting these together, we conclude that the transition from (M, k) to $(M, k+1)$ occurs at rate

$$Q_{(M,k)\to(M,k+1)} = \left(\frac{(n-k)r}{(h+km)r+(n-k)m}\right) \left(\frac{1}{h-1}\right) \left(\frac{1-r^{-1}}{1-r^{-m}}\right). \quad (83a)$$

Three other transitions are possible in the time-rescaled Markov chain, and their respective rates are obtained similarly:

$$Q_{(M,k)\to(R,k)} = \left(\frac{n-k}{(h+km)r+(n-k)m}\right) \left(\frac{1}{m-1}\right) \left(\frac{1-r}{1-r^{h}}\right) \quad (83b)$$

$$Q_{(R,k)\to(R,k-1)} = \left(\frac{k}{kmr+h+(n-k)m}\right) \left(\frac{1}{h-1}\right) \left(\frac{1-r}{1-r^{m}}\right) \quad (83c)$$

$$Q_{(R,k)\to(M,k)} = \left(\frac{kr}{kmr+h+(n-k)m}\right) \left(\frac{1}{m-1}\right) \left(\frac{1-r^{-1}}{1-r^{-h}}\right). \quad (83d)$$

In order to apply previous results of Hadjichrysanthou et al. [14], we must discretize the time-rescaled Markov chain. To do this, we compute the conditional probabilities of transition between each pair of states, conditioned

on leaving the current state:

$$P_{(M,k)\to(M,k+1)} = \frac{Q_{(M,k)\to(M,k+1)}}{Q_{(M,k)\to(M,k+1)} + Q_{(M,k)\to(R,k)}} = \frac{\left(\frac{1-r^{-h}}{h-1}\right)}{\left(\frac{1-r^{-h}}{h-1}\right) + r^{-h}\left(\frac{1-r^{-m}}{m-1}\right)} \tag{84a}$$

$$P_{(M,k)\to(R,k)} = \frac{Q_{(M,k)\to(R,k)}}{Q_{(M,k)\to(M,k+1)} + Q_{(M,k)\to(R,k)}} = \frac{r^{-h}\left(\frac{1-r^{-m}}{m-1}\right)}{\left(\frac{1-r^{-h}}{h-1}\right) + r^{-h}\left(\frac{1-r^{-m}}{m-1}\right)} \tag{84b}$$

$$P_{(R,k)\to(R,k-1)} = \frac{Q_{(R,k)\to(R,k-1)}}{Q_{(R,k)\to(R,k-1)} + Q_{(R,k)\to(M,k)}} = \frac{\left(\frac{1-r^{-h}}{h-1}\right)}{\left(\frac{1-r^{-h}}{h-1}\right) + r^{m}\left(\frac{1-r^{-m}}{m-1}\right)} \tag{84c}$$

$$P_{(R,k)\to(M,k)} = \frac{Q_{(R,k)\to(M,k)}}{Q_{(R,k)\to(R,k-1)} + Q_{(R,k)\to(M,k)}} = \frac{r^{m}\left(\frac{1-r^{-m}}{m-1}\right)}{\left(\frac{1-r^{-h}}{h-1}\right) + r^{m}\left(\frac{1-r^{-m}}{m-1}\right)}. \tag{84d}$$

For a Markov chain with this structure, the fixation probability from states $(M,0)$ and $(R,1)$, respectively, was derived by Hadjichrysanthou et al. [14]:

$$\rho_{(M,0)} = \frac{P_{(M,k)\to(M,k+1)}}{1 + \frac{x^n - x}{x-1} P_{(M,k)\to(R,k)}} \tag{85a}$$

$$\rho_{(R,1)} = \frac{P_{(R,k)\to(M,k)}}{1 + \frac{x^n - x}{x-1} P_{(M,k)\to(R,k)}}, \tag{85b}$$

with the quantity x is defined as

$$x = \frac{P_{(R,k)\to(R,k-1)}}{P_{(M,k)\to(M,k+1)}}. \tag{86}$$

Substituting from Eqs. (84a) and (84c), we have

$$x = \frac{\left(\frac{1-r^{-h}}{h-1}\right) + r^{-h}\left(\frac{1-r^{-m}}{m-1}\right)}{\left(\frac{1-r^{-h}}{h-1}\right) + r^{m}\left(\frac{1-r^{-m}}{m-1}\right)}. \tag{87}$$

To simplify the denominator in Eq. (85), we first compute from Eq. (87) that

$$\frac{1}{x-1} = \frac{\left(\frac{1-r^{-h}}{h-1}\right) + r^m \left(\frac{1-r^{-m}}{m-1}\right)}{\left(r^{-h} - r^m\right)\left(\frac{1-r^{-m}}{m-1}\right)}. \tag{88}$$

Substituting from Eqs. (84b), (87), and (88) and simplifying, the denominator in Eq. (85) becomes

$$\begin{aligned}
1 + \frac{x^n - x}{x-1} P_{(M,k)\to(R,k)} &= 1 + (x^{n-1} - 1)x \left(\frac{1}{x-1}\right) P_{(M,k)\to(R,k)} \\
&= 1 + (x^{n-1} - 1) \left(\frac{\left(\frac{1-r^{-h}}{h-1}\right) + r^{-h}\left(\frac{1-r^{-m}}{m-1}\right)}{\left(\frac{1-r^{-h}}{h-1}\right) + r^{m}\left(\frac{1-r^{-m}}{m-1}\right)}\right) \\
&\quad \times \left(\frac{\left(\frac{1-r^{-h}}{h-1}\right) + r^{m}\left(\frac{1-r^{-m}}{m-1}\right)}{\left(r^{-h} - r^m\right)\left(\frac{1-r^{-m}}{m-1}\right)}\right) \\
&\quad \times \left(\frac{r^{-h}\left(\frac{1-r^{-m}}{m-1}\right)}{\left(\frac{1-r^{-h}}{h-1}\right) + r^{-h}\left(\frac{1-r^{-m}}{m-1}\right)}\right) \\
&= 1 + (x^{n-1} - 1)\frac{r^{-h}}{r^{-h} - r^m} \\
&= 1 + \frac{x^{n-1} - 1}{1 - r^{m+h}} \\
&= \frac{x^{n-1} - r^{m+h}}{1 - r^{m+h}}.
\end{aligned}$$

To compute the overall fixation probability, we must consider the various locations the initial mutant may appear. We observe that, as $\epsilon \to 0$, the temperatures of all vertices converge to 1; thus the mutant type is equally likely to appear at any vertex. With probability $nm/(nm+h)$ it appears at an island vertex (connected or not). The mutation takes over the island with probability $(1-r^{-1})/(1-r^{-m})$, at which point we are in state $(R,1)$ of the slow timescale. With probability $h/(nm+h)$ the mutation arises at a hub vertex; at which point it has probability $(1-r^{-1})/(1-r^{-h})$ to take over the hub, resulting in state $(M,0)$. Putting this all together, we obtain

the overall fixation probability on the Cartwheel graph in the $\epsilon \to 0$ limit:

$$
\begin{aligned}
\rho_{CW_{n,m,h}}(r) &= \left(\frac{nm}{nm+h}\right)\left(\frac{1-r^{-1}}{1-r^{-m}}\right)\rho_{(R,1)} + \left(\frac{h}{nm+h}\right)\left(\frac{1-r^{-1}}{1-r^{-h}}\right)\rho_{(M,0)} \\
&= \frac{1-r^{-1}}{nm+h}\left(\frac{nm\rho_{(R,1)}}{1-r^{-m}} + \frac{h\rho_{(M,0)}}{1-r^{-h}}\right) \\
&= \frac{1-r^{-1}}{nm+h}\left(\frac{nmP_{(R,k)\to(M,k)}}{1-r^{-m}} + \frac{hP_{(M,k)\to(M,k+1)}}{1-r^{-h}}\right)\left(\frac{1-r^{m+h}}{x^{n-1}-r^{m+h}}\right) \\
&= \frac{\left(1-r^{-1}\right)\left(r^{m+h}-1\right)}{(nm+h)\left(r^{m+h}-x^{n-1}\right)} \\
&\quad \times \left(\frac{\binom{nm}{m-1}}{r^{-m}\binom{1-r^{-h}}{h-1} + \binom{1-r^{-m}}{m-1}} + \frac{\binom{h}{h-1}}{\binom{1-r^{-h}}{h-1} + r^{-h}\binom{1-r^{-m}}{m-1}}\right).
\end{aligned}
\tag{89}
$$

Note that, when $n = 1$, this expression is symmetric with regard to m and h. This makes sense because, if there is only one island, the hub and island are interchangeable.

7.3.2 Case $m = h$

In the case $m = h$, Eq. (87) simplifies to $x = r^{-m}$, and we have

$$
\begin{aligned}
\rho_{CW_{n,m,m}}(r) &= \frac{\left(1-r^{-1}\right)\left(r^{2m}-1\right)}{(nm+m)\left(r^{2m}-r^{-m(n-1)}\right)}\left(\frac{\binom{nm+m}{m-1}}{\left(1+r^{-m}\right)\binom{1-r^{-m}}{m-1}}\right) \\
&= \frac{\left(1-r^{-1}\right)\left(r^{2m}-1\right)}{\left(r^{2m}-r^{-m(n-1)}\right)\left(1+r^{-m}\right)\left(1-r^{-m}\right)} \\
&= \frac{\left(1-r^{-1}\right)\left(r^{2m}-1\right)}{\left(r^{2m}-r^{-m(n-1)}\right)\left(1-r^{-2m}\right)} \\
&= \frac{1-r^{-1}}{\left(r^{2m}-r^{-m(n-1)}\right)r^{-2m}} \\
&= \frac{1-r^{-1}}{1-r^{-(mn+m)}}.
\end{aligned}
$$

This is equal to the fixation probability $\rho_{K_{mn+m}}(r)$ for the complete graph K_{mn+m}. Thus, for $h = m$ and $\epsilon \to 0$, the Cartwheel has the same fixation probability as a well-mixed population of the same size.

7.3.3 Weak selection with $\epsilon \to 0$

With the aid of Mathematica, we compute the Taylor expansion of Eq. (89):

$$\rho_{CW_{n,m,h}}(1+\delta) = \frac{1}{nm+h} + \delta\rho' + \mathcal{O}(\delta^2), \tag{90}$$

with

$$\rho' = \frac{N-1}{2N} + \frac{mnh(h-m)\big(m(n-2)(h-1)+h(m-1)\big)}{2\big(h(m-1)+m(h-1)\big)(mn+h)\big(mn(h-1)+h(m-1)\big)}. \tag{91}$$

We observe that, for the Cartwheel $CW_{n,m,h}$ in the $\epsilon \to 0$ limit, the neutral fixation probability, $\rho^\circ = 1/(nm+h) = 1/N$, is the same as a well-mixed population. Meanwhile, the second term of ρ' has the sign of $h-m$. We conclude that the Cartwheel $CW_{n,m,h}$ (in the $\epsilon \to 0$ limit) is an amplifier (both uniform and relative) of weak selection for $h > m$, and a suppressor (uniform and relative) of weak selection for $h < m$. For $h = m$, $CW_{n,m,h}$ has the same fixation probability as the complete graph, K_{mn+h}, in the $\epsilon \to 0$ limit, as was shown in the previous subsection,

Eq. (91) shows numerical agreement with our solution for ρ' in Eq. (82) when evaluated for small ϵ. Thus the ordering of the $\epsilon \to 0$ limit and the δ-derivative appears not to matter; however, we have not proven this formally.

In the case $m = 2$ and $h = n$, Eq. (91) becomes

$$\rho' = \frac{(n-1)(2n+1)\,(2n^2+n+2)}{6n(2n-1)(3n-2)}. \tag{92}$$

As $n \to \infty$ (noting that $N = 3n$ for $h = n$, $m = 2$), we have $\rho' \sim N/27$ and $\rho'/(N\rho^\circ) \sim N/27$. Since the Star S_n has $\rho'/(N\rho^\circ) \to 1$ in the $n \to \infty$ limit, the Cartwheel graph $CW_{n,2,n}$ (with sufficiently small ϵ) must eventually surpass the Star in as a relative amplifier of weak selection (i.e., the Cartwheel must eventually have a greater $\rho'/(N\rho^\circ)$ ratio than the Star). Numerical computation shows that this first happens for size $N = 21$.

7.3.4 Limit of many islands

The formula for $\rho(r)$, Eq. (89), simplifies greatly in the limit of many islands ($n \to \infty$). Since each island is connected to a separate hub vertex, the number of hub vertices must also increase n. We therefore set $h = kn$ for

arbitrary $k \geq 1$, so that the number of hub vertices scales linearly with the number of islands.

First we consider the case $r < 1$. Rewriting x from Eq. (87) as

$$x = \frac{r^{-h}\left(1 + (h-1)\left(\frac{1-r^{-m}}{m-1}\right)\right) - 1}{r^{-h}\left(1 + (h-1)r^{h+m}\left(\frac{1-r^{-m}}{m-1}\right)\right) - 1}, \tag{93}$$

we can see that x behaves asymptotically as $r^{-(h+m)}$ as $h \to \infty$, in the sense that

$$\lim_{h \to \infty} \frac{x}{r^{-(h+m)}} = 1. \tag{94}$$

Substituting $r^{-(h+m)}$ for x in Eq. (89), letting $h = kn$, and taking $n \to \infty$, we have (still in the $r < 1$ case),

$$\begin{aligned}
\lim_{n \to \infty} \rho_{CW_{n,m,h}}(r) &= \lim_{n \to \infty} \frac{\left(1 - r^{-1}\right)\left(r^{m+kn} - 1\right)}{\left(n(m+k)\right)\left(r^{m+kn} - r^{-(m+kn)(n-1)}\right)} \\
&\times \left(\frac{\left(\frac{nm}{m-1}\right)}{r^{-m}\left(\frac{1-r^{-kn}}{kn-1}\right) + \left(\frac{1-r^{-m}}{m-1}\right)} + \frac{\left(\frac{kn}{kn-1}\right)}{\left(\frac{1-r^{-kn}}{kn-1}\right) + r^{-kn}\left(\frac{1-r^{-m}}{m-1}\right)} \right) \\
&= 0.
\end{aligned}$$

Thus, for a deleterious mutation, the probability of fixation vanishes in the limit of many islands.

For $r > 1$, we have from Eq. (87) that $\lim_{h \to \infty} x = 0$. Substituting 0 for x, letting $h = kn$, and taking $n \to \infty$, we obtain

$$\begin{aligned}
\lim_{n \to \infty} \rho_{CW_{n,m,h}}(r) &= \lim_{n \to \infty} \frac{\left(1 - r^{-1}\right)\left(r^{m+kn} - 1\right)}{n(m+k)r^{m+kn}} \\
&\times \left(\frac{\left(\frac{nm}{m-1}\right)}{r^{-m}\left(\frac{1-r^{-kn}}{kn-1}\right) + \left(\frac{1-r^{-m}}{m-1}\right)} + \frac{\left(\frac{kn}{kn-1}\right)}{\left(\frac{1-r^{-kn}}{kn-1}\right) + r^{-kn}\left(\frac{1-r^{-m}}{m-1}\right)} \right) \\
&= \frac{1 - r^{-1}}{m+k}\left(\frac{m}{1 - r^{-m}} + k\right) \\
&= \left(\frac{m + k(1 - r^{-m})}{m+k}\right)\frac{1 - r^{-1}}{1 - r^{-m}}.
\end{aligned}$$

Putting these cases together, we have the following result for fixation probability on the Cartwheel graph when first $\epsilon \to 0$ and then $n \to \infty$:

$$\lim_{n\to\infty} \rho_{CW_{n,m,h}}(r) = \begin{cases} 0 & r \leq 1 \\ \left(\dfrac{m + k(1 - r^{-m})}{m + k}\right) \dfrac{1 - r^{-1}}{1 - r^{-m}} & r > 1. \end{cases} \tag{95}$$

This limiting fixation probability is discontinuous at $r = 1$. Indeed, if we take the limit as r approaches 1 from the positive side, we have

$$\lim_{r\to 1^+} \lim_{n\to\infty} \rho_{CW_{n,m,h}}(r) = \frac{1}{k+m}. \tag{96}$$

Thus, the fixation probability jumps from 0 to $1/(k+m)$ as r increases past 1.

The largest fixation probability in Eq. (95) arises for $k = 1$ (meaning $h = n$) and $m = 2$. This corresponds to a "spider" structure (Fig 9B of the main text). In this case, we have

$$\lim_{n\to\infty} \rho_{CW_{n,m,h}}(r) = \begin{cases} 0 & r \leq 1 \\ \dfrac{1 - r^{-2}/3}{1 + r^{-1}} & r > 1. \end{cases} \tag{97}$$

References

[1] Maciejewski W. Reproductive value in graph-structured populations. Journal of Theoretical Biology. 2014;340:285–293.

[2] Tarnita CE, Taylor PD. Measures of relative fitness of social behaviors in finite structured population models. The American Naturalist. 2014;184(4):477–488.

[3] Allen B, Lippner G, Chen YT, Fotouhi B, Momeni N, Yau ST, et al. Evolutionary dynamics on any population structure. Nature. 2017;544(7649):227–230.

[4] Allen B, Sample C, Dementieva Y, Medeiros RC, Paoletti C, Nowak MA. The Molecular Clock of Neutral Evolution Can Be Accelerated or Slowed by Asymmetric Spatial Structure. PLOS Computational Biology. 2015;11(2):e1004108. doi:10.1371/journal.pcbi.1004108.

[5] Allen B, McAvoy A. A mathematical formalism for natural selection with arbitrary spatial and genetic structure. Journal of Mathematical Biology. 2019;doi:10.1007/s00285-018-1305-z.

[6] McAvoy A, Allen B. Fixation probabilities in evolutionary dynamics under weak selection. Journal of Mathematical Biology. 2021;Forthcoming. Available from: https://arxiv.org/abs/1908.03827.

[7] Möller M, Hindersin L, Traulsen A. Exploring and mapping the universe of evolutionary graphs identifies structural properties affecting fixation probability and time. Communications Biology. 2019;2(1):137.

[8] Cuesta FA, Sequeiros PG, Rojo ÁL. Suppressors of selection. PLOS One. 2017;12(7):e0180549.

[9] Cuesta FA, Sequeiros PG, Rojo ÁL. Evolutionary regime transitions in structured populations. PLOS One. 2018;13(11):e0200670.

[10] Voorhees B, Murray A. Fixation probabilities for simple digraphs. Proceedings of the Royal Society A: Mathematical, Physical and Engineering Science. 2013;469(2154):20120676.

[11] Monk T, Green P, Paulin M. Martingales and fixation probabilities of evolutionary graphs. Proceedings of the Royal Society A: Mathematical, Physical and Engineering Science. 2014;470(2165):20130730.

[12] Sample C, Allen B. The limits of weak selection and large population size in evolutionary game theory. Journal of Mathematical Biology. 2017;75(5):1285–1317.

[13] Allen B, Sample C, Jencks R, Withers J, Steinhagen P, Brizuela L, et al. Transient amplifiers of selection and reducers of fixation for death-Birth updating on graphs. PLOS Computational Biology. 2020;16(1):e1007529.

[14] Hadjichrysanthou C, Broom M, Rychtář J. Evolutionary games on star graphs under various updating rules. Dynamic Games and Applications. 2011;1(3):386.

[15] Yin G, Zhang Q. Discrete-time Markov chains: two-time-scale methods and applications. vol. 55. Springer Science & Business Media; 2005.
